# Integrative analysis of lysine acetylation-related genes and identification of a novel prognostic model for oral squamous cell carcinoma

**DOI:** 10.3389/fmolb.2023.1185832

**Published:** 2023-08-29

**Authors:** Shi-Zhou Deng, Xuechen Wu, Jiezhang Tang, Lin Dai, Bo Cheng

**Affiliations:** ^1^ Department of Hepatobiliary Surgery, Xi-Jing Hospital, The Fourth Military Medical University, Xi’an, China; ^2^ Department of Stomatology, Zhongnan Hospital of Wuhan University, Wuhan, China; ^3^ Department of Burn and Plastic Surgery, Tangdu Hospital, Fourth Military Medical University, Xi’an, China; ^4^ Department of Stomatology, The First Hospital of Wuhan, Wuhan, China

**Keywords:** OSCC, oral squamous cell carcinoma, lysine acetylation, prognostic model, TCGA, GEO

## Abstract

**Introduction:** Oral squamous cell carcinoma (OSCC), which accounts for a high proportion of oral cancers, is characterized by high aggressiveness and rising incidence. Lysine acetylation is associated with cancer pathogenesis. Lysine acetylation-related genes (LARGs) are therapeutic targets and potential prognostic indicators in various tumors, including oral squamous cell carcinoma. However, systematic bioinformatics analysis of the Lysine acetylation-related genes in Oral squamous cell carcinoma is still unexplored.

**Methods:** We analyzed the expression of 33 Lysine acetylation-related genes in oral squamous cell carcinoma and the effects of their somatic mutations on oral squamous cell carcinoma prognosis. Consistent clustering analysis identified two lysine acetylation patterns and the differences between the two patterns were further evaluated. Least absolute shrinkage and selection operator (LASSO) regression analysis was used to develop a lysine acetylation-related prognostic model using TCGA oral squamous cell carcinoma datasets, which was then validated using gene expression omnibus (GEO) dataset GSE41613.

**Results:** Patients with lower risk scores had better prognoses, in both the overall cohort and within the subgroups These patients also had “hot” immune microenvironments and were more sensitive to immunotherapy.

**Disscussion:** Our findings offer a new model for classifying oral squamous cell carcinoma and determining its prognosis and offer novel insights into oral squamous cell carcinoma diagnosis and treatment.

## 1 Introduction

Oral squamous cell carcinoma (OSCC) is one of the most common cancers worldwide and accounted for around 369,000 new in 2012. OSCC incidence has continued to grow, with two-thirds of the cases occurring in developing countries. OSCC has a wide range of clinical patterns (Ghantous and Abu Elnaaj, 2017), and the majority of the cases are associated with lifestyle habits like smoking, excessive alcohol consumption, and betel nut chewing. According to the National Comprehensive Cancer Network (NCCN) clinical practice guidelines in oncology, all OSCC is primarily treated through surgery in combination with radiotherapy and chemotherapy, and the use of targeted treatments is recommended for advanced cases (stages III–IV) (Warnakulasuriya, 2009). Following initial surgery and proper adjuvant treatment, the pathologic nodal stage is the main predictor of the malignant degree of OSCC patients (Zanoni et al., 2019). However, OSCC recurrence is common after the first R0 resection, resulting in a low survival rate (Warnakulasuriya, 2009), with an inadequate quality of life (Lin et al., 2022).Moreover, survival rates decline with increasing time before treatment initiation (Jensen et al., 2021). These factors emphasize the need for early OSCC diagnosis as well as novel molecular targets for treatment. For instance, although cetuximab, which targets the epidermal growth factor receptor, was approved for OSCC treatment in 2006 and anti-PD1 therapy has recently been used to treat patients with metastatic disease following relapse or progression during or after chemotherapy (Ferris et al., 2016), their efficacies have not been significant. Thus, understanding the molecular changes that underlie OSCC pathogenesis and the factors that contribute to OSCC patient prognosis is an unmet medical need.

Cell transporter functional expression has been demonstrated to be modulated by post-translational modification (PTM) via a variety of molecular pathways. These changes are made by adding These changes are made by adding specific chemical groups to certain amino acid residues (Czuba et al., 2018). Acetylation is a common PTM initiated by specific enzymes that transfer acetyl groups to the amino side chain of lysine. Recent studies show that acetylation can also occur non-enzymatically and is influenced by the availability of acetyl-CoA (Narita et al., 2019). Although acetylation was previously thought to be specific to histones, thousands of non-histone proteins have been shown to contain lysine acetylation, including nuclear, mitochondrial, and cytoplasmic proteins. Non-histone acetylation regulates several cellular processes, including transcription, DNA damage repair, and cell signaling. Lysine acetylation drives tumorigenesis by actively modifying the expression and function of oncogenic or tumor-suppressive factors (O’Garro et al., 2021; Hu et al., 2022). The acetylation process can influence tumor formation and progression by modulating immune activity and response in a variety of ways. Several immune-related acetylation/deacetylation modification targets are mentioned below (Ding et al., 2022). For example, p300 can acetylates PD-L1 and inhibits its translocation into the nucleus (Gao et al., 2020). And in non-small cell lung cancer HDAC3 can be suppressed by the decreased COP1, which increases PD-L1 expression (Wang H. et al., 2020).

Histone and non-histone acetylation, have double-edged roles in tumor metastasis and metabolism (Hu et al., 2022). Four human histone deacetylase inhibitors (HDACi) with the potential to trigger tumor suppressor genes, have emerged as epigenome-targeting drugs that can improve the chemotherapeutic and radiosensitivity of cancer cells, and have received FDA approval for use in clinical settings (Ding et al., 2022). DLUE1 is reported to be overexpressed in early OSCC tumors, and its knockdown suppresses OSCC cell proliferation, migration, and invasion, implying that DLEU1 drives the expression of several genes during OSCC carcinogenesis (Hatanaka et al., 2021). The expression of the deacetylase genes, HDAC6 and HDAC9, is markedly elevated in OSCC (Sakuma et al., 2006; Rastogi et al., 2016). Antitumor effects of novel HDACi in OSCC have also been reported (Bai et al., 2011). For instance, HDACi target cancer stem cells by inhibiting tumor growth and inducing cytotoxicity and intracellular reactive oxygen species and are potential OSCC treatments (Marques et al., 2020). Impairment of lysine acetylation is thought to impair ribosome biogenesis and might contribute to OSCC pathogenesis (Dong et al., 2022).

In this study, we used bioinformatics to analyze the expression of 33 lysine acetylation-related genes (LARGs) as well as their mutations in OSCC tissues vs. normal tissues and then validated their expression using RT-qPCR. Based on the expression of “HDAC3” and “SIRT5”, OSCC patients were divided into two groups, and their correlation with clinical characteristics examined. Univariate and LASSO regression analyses were used to develop an OSCC prognostic model. The efficacies of immunotherapy and chemotherapy, as well as the OSCC immune landscape, were analyzed in various risk groups.

## 2 Materials and methods

### 2.1 Oral squamous cell carcinoma patient datasets

RNA sequencing (RNA-seq) data on tissues from 323 OSCC patients and 32 normal tissues, as well as associated clinical data, were downloaded from TCGA. Gene microarray data and associated clinical data for 97 tumor samples were obtained from dataset GSE41613 from gene expression omnibus (GEO) (Supplementary Table S1). The “limma” package was used for internal standards and then applied to perform difference analysis.

### 2.2 Identification of differentially expressed lysine acetylation-related genes (LARGs)

Thirty-three LARGs were retrieved from a previous review (Narita et al., 2019) (Supplementary Table S2). The “limma” package was used to identify differentially expressed LARGs with *p* < 0.05. Next, we evaluated gene express variations in the 33 LARGs in each TCGA OSCC sample to identify the LARGs associated with mutagenesis. Data on gene mutations was also gathered from TCGA. The frequency of different mutations was computed. Finally, the R package “maftools” was used for visualization. Waterfall diagrams were used to visualize the status of somatic mutation integration in OSCCs. Univariate analysis was used to identify prognostic LARGs. Protein–protein interaction (PPI) networks for the 30 connected LARGs were constructed on STRING (https://cn.string-db.org/) (von Mering et al., 2005).

### 2.3 mRNA and protein level analyses of OSCC samples

This study involved patients who underwent routine intraoral examination, followed by oral mucosal biopsy and diagnosis of squamous cell carcinoma of the oral cavity. Ten pairs of OSCC and adjacent normal tissues were collected at Zhongnan Hospital. Patients with a history of systemic illness or with other primary tumors were excluded from the analysis. OSCC samples and matched adjacent noncancerous tissues were obtained before preoperative radiotherapy or chemotherapy and immediately frozen in liquid nitrogen, followed by storage at −80°C until RNA extraction. Total RNA was extracted using Trizol reagent (Servicebio, China). Ethical approval for the study (No. 2022095K) was granted by Zhongnan Hospital of Wuhan University Medical Ethics Committee. RT-qPCR was done on a BIO-RAD system using a SYBR green dye qPCR mix (Servicebio, China). Primer information is provided in Supplementary Table S3. The paired-T test was used to determine the expression levels of the LARGs and GAPDH. Human Protein Atlas (HPA) immunohistochemistry data were used to identify the protein levels of two patterns, SIRT5 and HDAC3, in paracancerous tissue and malignant tissues.

### 2.4 Consensus clustering analysis of the LARGs

The “ConsensuClusterPlus” package was used to delimit distinct lysine acetylation-related OSCC patterns (Seiler et al., 2010). Based on different lysine acetylation-associated OSCC patterns, we examined the clinicopathological features and prognosis of the patients. The Kaplan–Meier (KM) analysis of the correlation between the lysine acetylation-associated OSCC patterns was carried out by R packages “survival” and “survminer” (Rich et al., 2010).

### 2.5 Identification of a LARGs prognostic signature for OSCC

GSE41613 was used as the test cohort, whereas the TCGA dataset was used as the training cohort. The LARGs-associated signature was used to set up the prognostic model in the training cohort. Next, univariate Cox regression analysis was used to identify the prognostic differentially expressed genes (DEGs) between the lysine acetylation-related patterns. LASSO regression analysis was then used to identify prognostic DEGs (*p* < 0.05) using the “glmnet” package (Simon et al., 2011). The risk score of the patients was calculated by the formula as follows: $Risk\;score=\sum_{i=1}^{n}coefi*expi.$ The median risk score was used to group the patients. Survival differences between the two groups were comparatively analyzed through KM survival analysis. Based on gene expression, principal component analysis (PCA) was done with the “stats” package. Moreover, t-distributed stochastic neighbor embedding (t-SNE) was conducted to discuss the distribution of different groups via the “Rtsne” package. The receiver operating characteristic (ROC) curve analyses were carried out to estimate the prognostic power of the gene signature by using the “survivalROC” package. The prognostic relationship between risk score and age, gender, grade, clinical stage, and immune score was analyzed. Additionally, we explored the correlation between risk scores and cluster patterns.

### 2.6 Construction of the OSCC nomogram

We created a nomogram based on the risk scores and the clinical data of the OSCC patients, including age, stage, grade, and genderto expoit the predictive value of the eight-gene-based signature for clinical application. To this end, the ‘rms’, ‘nomogramEx’, and ‘regplot’ R packages were used to construct the nomogram. Next, ROC curve analysis was used to assess how well the nomogram could predict OSCC prognosis (Pencina and D'Agostino, 2004). Additionally, we used calibration curves to determine if the projected survival outcome (one-, three-, and five-year survival) was close to the actual outcome (Alba et al., 2017). The 45° line shows the best nomogram-predicted survival.

### 2.7 Validation of grouping efficacy and association analysis of immune cell infiltration

The relationship between risk scores and immune cells infiltration in OSCC samples was analyzed by the Pearson correlation analysis using the GSVA package. Statistical analysis was done using the ssGSEA algorithm (Hänzelmann et al., 2013). Various immune indicators to study the relationship between factors and immune phenotypes. We analyzed the association between risk scores and immune cell infiltration, as well as the expression of immune biomarkers, HLA family, chemokines, and chemokine receptors. Immune checkpoint was examined via Pearson correlation analysis using *p* = 0.05 as the cutoff threshold. The immunophenotype scores (IPS) of the patients were used to predict OSCC response to checkpoint blockade immunotherapy (Charoentong et al., 2017).

### 2.8 Drug sensitivity analysis

To assess the therapeutic potential of chemotherapy drugs on OSCC, the semi-inhibitory concentration (IC50) of common drugs was determined using the “pRRophetic” package (Geeleher et al., 2014). The sensitivity of the chemotherapeutic agents in different patient groups was also predicted.

### 2.9 Statistical analysis

Statistics acquired from TCGA were merged and conducted on R then processed and analyzed on R using the indicated packages. Normally distributed continuous variables were expressed as Mean ± standard deviation. Non-normally distributed continuous variables were presented as medians (range). Categorical variables were described as counts and percentages. Two-sided *p* < 0.05 indicated statistically significant differences.

## 3 Results

### 3.1 The landscape of lysine acetylation-related genes in OSCC patients

**FIGURE 1:**
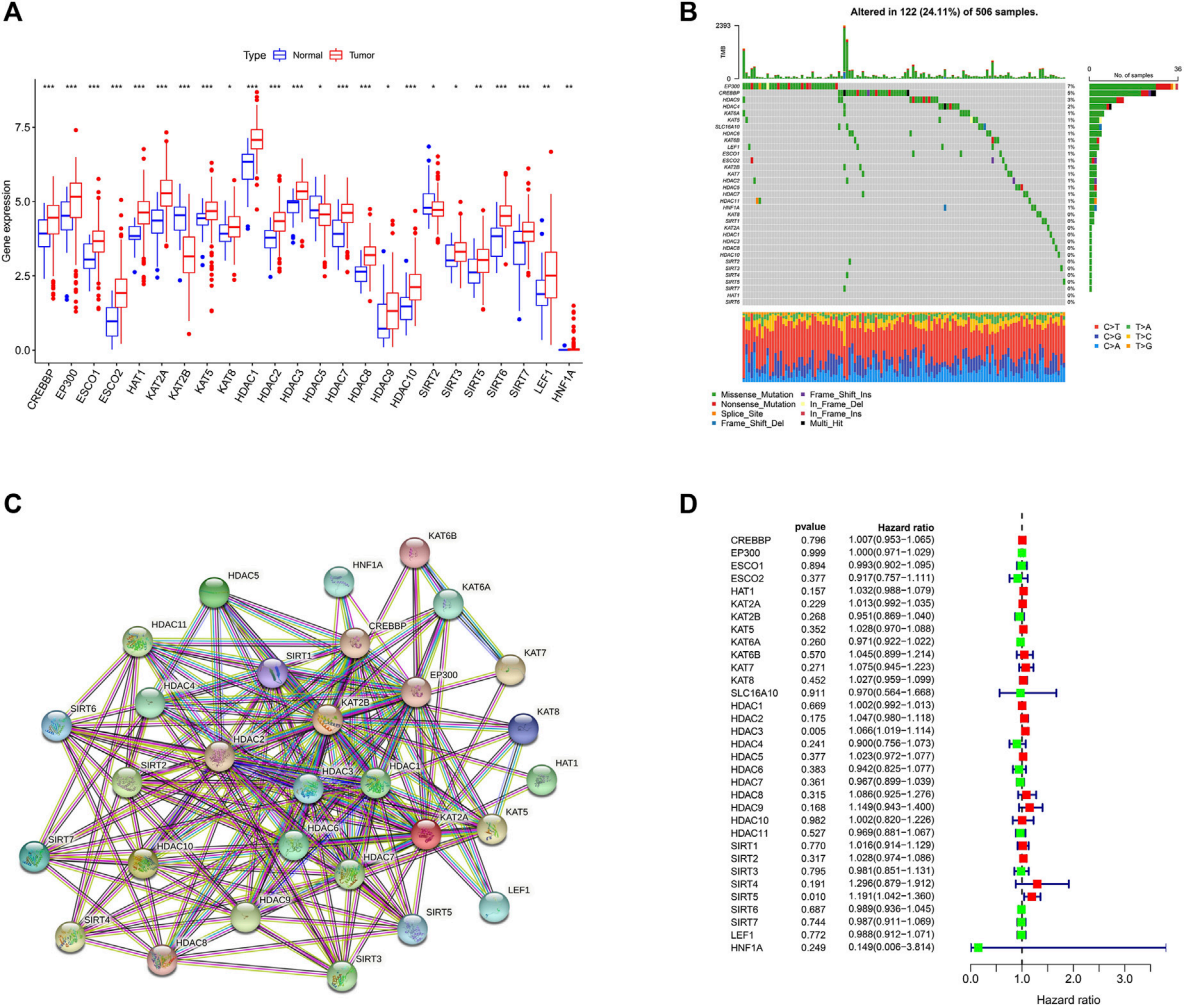
Landscape of (lysine acetylation-related genes) LARGs in OSCC **(A)** Molecular expression of LARGs in normal tissues compared with oral squamous cell carcinomas. **(B)** The genetic alterations of LARGs in OSCC. **(C)** The interactions between the candidate genes were shown by the PPI network. **(D)** The findings of the univariate Cox regression demonstrate the relationship between OS and gene expression.

The detailed flowchart of the study is shown in Supplementary Figure S1. Using the TCGA dataset, we identified the expression levels of 33 LARGs in OSCC samples, and normal paracancerous specimens and found that the 24 of the 33 LARGs (73%) were expressed significantly different in OSCCs (Figure 1A).

Given the importance of gene mutations in carcinogenesis, we investigated the somatic mutations of 33 LARGs in OSCC samples and found that 122 of the 506 (24.11%) OSCC samples we analyzed had genetic changes. Among the LARGs we studied, EP300 was shown to have the highest mutation rate, followed by CREBBP and HDAC9. (Figure 1B). EP300 and CREBBP are both often mutated in squamous cell carcinoma and lymphomas (Attar and Kurdistani, 2017). Most of the changes are missense point mutations. HDAC9 interacts with a variety of transcriptional repressors and oncogenes (Ning et al., 2020) and may influence anticancer immune responses by limiting T-cell infiltration into the tumor microenvironment (TME) (Yang et al., 2021).

A PPI network revealed that 30 LARGs were closely interconnected (Figure 1C), the other 3 genes were eliminated because they do not interact with other lysine acetylation-related genes. Univariate Cox regression analysis revealed that high HDAC3 and SIRT5 expression was associated with poor OSCC survival (Hazard ratio, HR: >1; Figure 1D).

### 3.2 HDAC3 and SIRT5 are upregulated in OSCC tissues when compared with normal tissue

**FIGURE 2:**
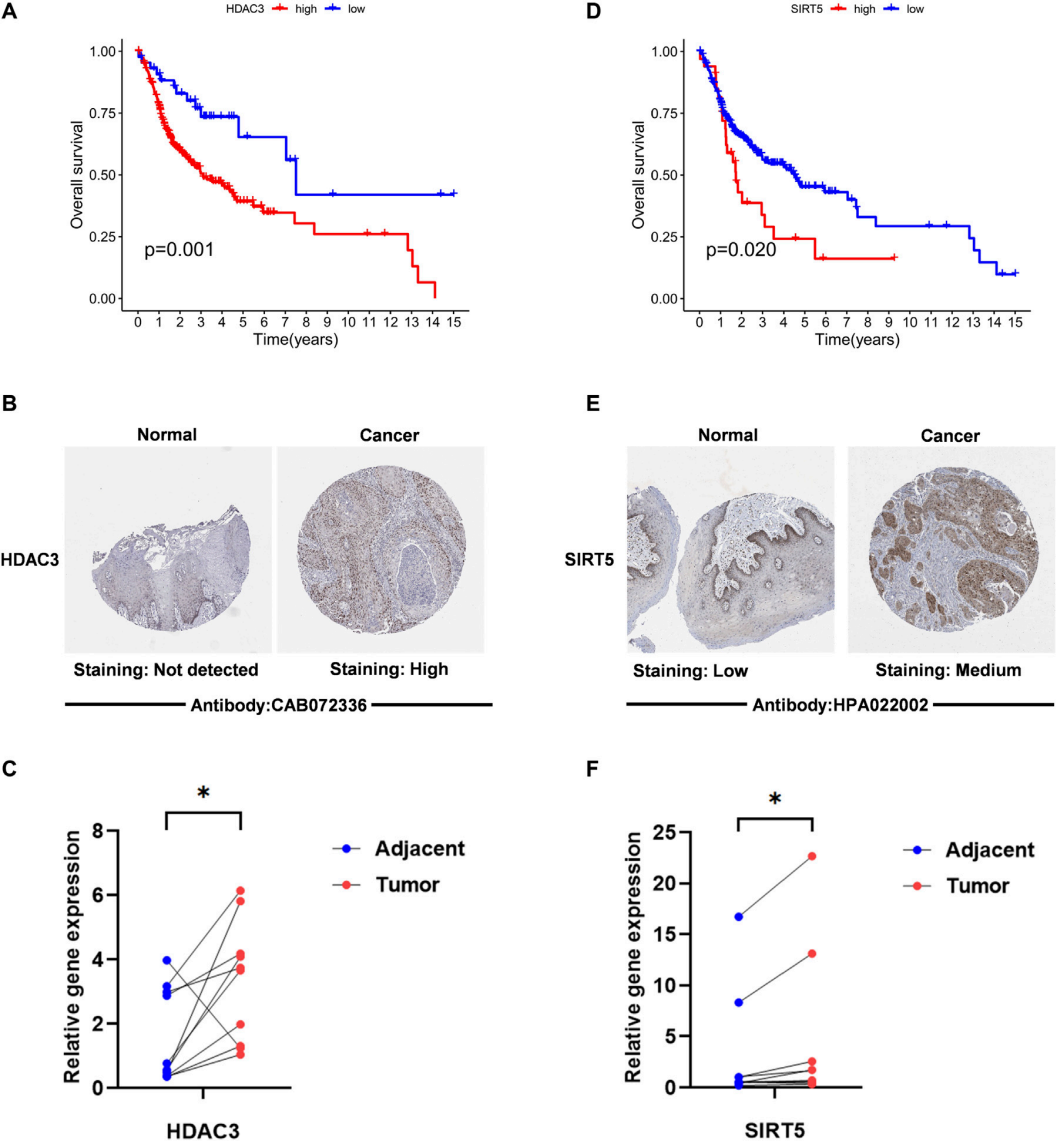
The expression level of “HDAC3” “SIRT5” in OSCC. **(A,D)** Kaplan–Meier survival analysis based on the expression of acetylation-related genes The OSCC patient survival curve for those with high and low gene expression was depicted by the red curve and the blue curve. **(B,E)** The HPA immunohistochemistry data were utilized to identify the protein levels of two genes in normal and malignant tissues. **(C,F)** HDAC3 and SIRT5 expression levels in OSCC tissues and surrounding normal tissues are compared. RT-PCR was used to identify the alterations in the expression of 2 LARGs in OSCC and its normal tissue. *if *p* < 0.05, ** if *p* < 0.01, and *** if *p* < 0.001.

We next conducted studies based on the expression of HDAC3 and SIRT5, and it appeared that there were substantial disparities in their overall survival (Figures 2A, D). Analysis of immunohistochemical data on HPA revealed that OSCC tissues exhibited significantly higher SIRT5 and HDAC3 staining when compared with normal tissues (Figures 2B, E). Moreover, RT-qPCR analysis revealed that SIRT5 and HDAC3 expression levels in cancer tissues were significantly higher than in normal tissues (*p* < 0.05; Figures 2C, F).

### 3.3 Tumor classification based on the prognostic value of lysine acetylation regulators

**FIGURE 3:**
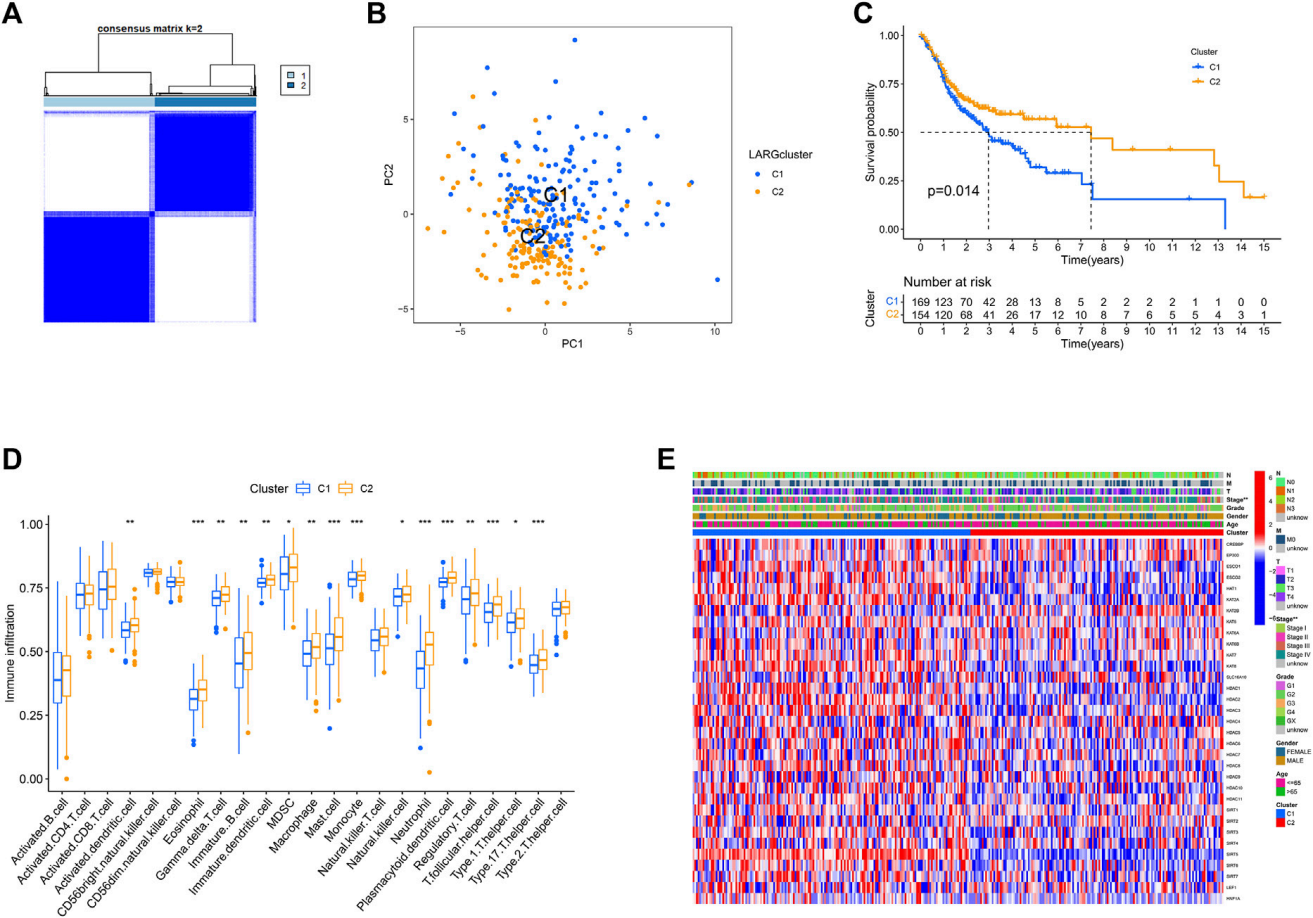
Consensus clustering is carried out based on the LARGs. **(A)** The consensus clustering was used to divide 323 OSCC patients into two groups (k = 2). **(B)** PCA analysis showed a clear distinction between the cluster 1 and 2. **(C)** Survival curves for genes involved in RNA methylation that are linked to overall survival. Clusters 1 and 2 were shown to be substantially linked to survival (*p* = 0.014). **(D)** 23 immune cell types infiltration is significantly different in two clusters. **(E)** The clinicopathologic characteristics between the two clusters are shown on a heatmap.

Consistent clustering was used to examine SIRT5 and HDAC3 expression in a TCGA dataset of 323 OSCC cases. To this end we grouped the OSCC patients into two clusters based on cumulative distribution function (CDF) values (k = 2; Figure 3A, and k = 3–9; Supplementary Figure S2). PCA analysis found that the two clusters are clearly identifiable (Figure 3B).

This analysis also revealed that overall survival of cluster 1 was worse than that of cluster 2 (*p* = 0.014; Figure 3C). Analysis of whether the variability in survival was caused by differences in infiltration by the 23 immune cells in the 2 clusters revealed that immune cell infiltration differed significantly in 16 of 23 OSCCs (Figure 3D). These findings suggest that in the context of reduced expression of lysine acetylation-associated genes, OSCC patients with immune cell infiltration had better prognosis.

Furthermore, except for stage, other clinical parameters, including grade, gender, age, and TNM did not differ across these two clusters In cluster 1, most genes are upregulated, while in cluster 2, the genes are downregulated, as shown in the heat map (Figure 3E).

### 3.4 Developing an independent prognostic risk model based on LARGs clustering

**FIGURE 4:**
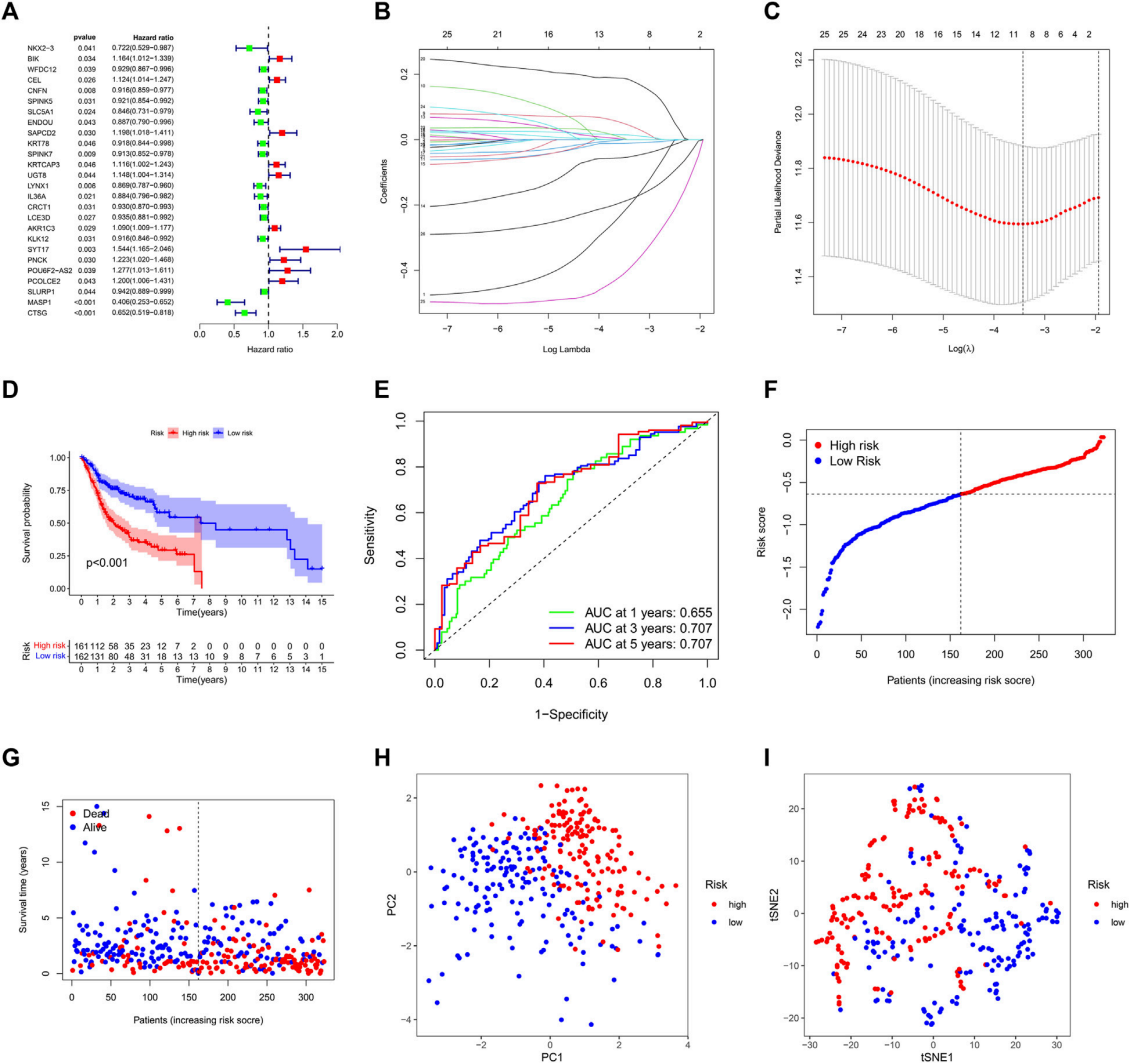
Construction of the lysine acetylation-related prognostic signature in the training cohort. **(A)** An investigation of OS for each DEG on clusters 1 and 2 using univariate cox regression. **(B)** LASSO regression of DEGs in OSCC. **(C)** Cross validation in the LASSO regression. **(D)** The OS of OSCC patients in the high-risk group was considerably poorer than that of the low-risk group, according to K-M curves. **(E)** An evaluation of the prognostic signature for OS in OSCC patients using ROC curves. **(F,G)** OSCC patients characteristics by high- and low-risk categories **(H,I)** To demonstrate how the samples of the various risk groups associated with lysine acetylation were dispersed independently, PCA **(H)** and t-SNE **(I)** were used.

We used “limma” package of R (4.1.1) to conduct, we discovered 323 DEGs between the two clusters (Supplementary Table S4), these DEGs were then examined via univariate Cox regression analysis. Twenty-six genes were finally proved that can be employed as distinct prognostic indicators (Figure 4A). After filtration, LASSO Cox regression analysis found NKX2-3, SAPCD2, SPINK7, LYNX1, AKR1C3, SYT17, MASP1, and CTSG to be significantly associated with overall survival (OS) (Figures 4B, C; adjusted *p* < 0.05).

The genes were used to calculate risk score based on the formula:

$$\begin{array}{ll} risk\;score & =\left(-0.207\;*\;NKX2-3\right)+\left(0.045\;*\;SAPCD2\right)+\left(-0.011\;*\;SPINK7\right)+\left(-0.054\;*\;LYNX1\right)\\ & +\left(0.014\;*\;AKR1C3\right)+\left(0.185\;*\;SYT17\right)+\left(-0.399\;*\;MASP1\right)+\left(-0.185\;*\;CTSG\right). \end{array}$$

Next, samples were divided into the high and low survival risk groups based on the median risk score, as shown using KM survival curves (*p* < 0.001). These analyses indicate that the multigene signature had a significant prognostic value (Figure 4D) and that the risk scores distinguished patients with high and low survival rates (Figure 4F). The area under the curve (AUC) analysis at one, three, and 5 years (AUC: 0.655, 0.707, and 0.707, respectively) showed that the prognostic signature was highly accurate at predicting OS in OSCC patients (Figure 4E). PCA analysis and t-SNE analysis suggested that the OSCCs in distinct risk categories were distributed in two directions (Figures 4G, H).

### 3.5 Validation of the prognostic value in the subgroups

Next, we split the GEO dataset into two categories based on risk score (Supplementary Figure S3). KM (*p* = 0.02; Supplementary Figure S3A) and ROC curve analyses revealed that the low-risk group had a higher overall survival rate, indicating that the model was accurate (one-three-, and five-year AUC: 0.736, 0.645, and 0.661, respectively; Supplementary Figure S3B). There were fewer deaths in the low-risk group, which exhibited lower expression levels of the risk genes (Supplementary Figures S3C, F). Finally, t-SNE analysis and PCA revealed that the risk genes were very effective in differentiating the two risk groups (Supplementary Figures S3D–E).

### 3.6 Subgroup survival analysis based on clinical parameters

To determine the ability of various clinical parameters to predict OSCC prognosis, we carried out a stratified analysis of clinical parameters in the test cohort by creating multiple subgroups for the patients in the TCGA dataset using various clinical parameters. KM analysis of the correlation between age (≤65 and >65 years), sex, grade (G1–G2 or G3–G4), stage (I–II or III–IV), and survival indicated that except for G3–G4, high-risk patients had a lower likelihood of survival than low-risk patients (Supplementary Figures S4A–H). We also studied how the clinical parameters and the risk scores correlated with one another. This analysis revealed that high-risk scores and the AJCC stage, clusters, and immune scores differ significantly from each other (Supplementary Figures S5A–F). High-risk scores were mainly observed in patients with lower immune scores when compared with those with high immune scores. Advanced disease stage was also associated with higher risk scores.

### 3.7 Development of a nomogram and model efficiency prediction

**FIGURE 5:**
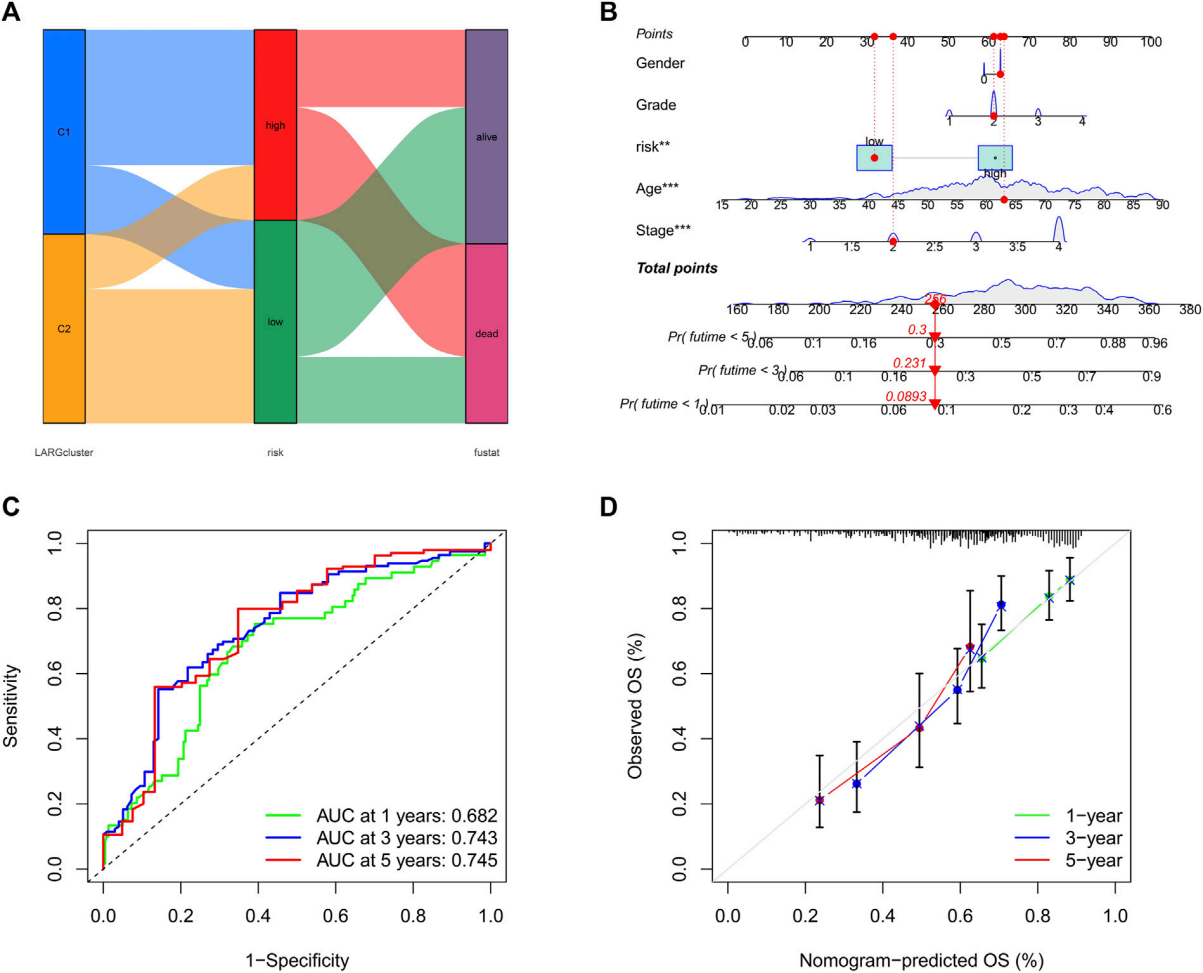
**(A)** Sankey plot shows quantities of patients flow from 2 clusters to the risk score distributions group than to final status. **(B)** Nomogram for OSCC patient survival predictions at 1, 3, and 5 years **(C)** The ROC curves for 1−, 3−, and 5−year OS in OSCC patients. **(D)** Calibration curves of the nomogram measured by Hosmer-Lemeshow test.

Sankey plot analysis revealed that the patients were distributed into two LARG clusters, two risk score clusters, and two future status clusters (Figure 5A).

Next, we developed a nomogram to illustrate the connection between these independent prognostic markers and survival probabilities (Figure 5B). Clinicians might forecast a patient’s prognosis based on their total points. Patients with higher total points had lower survival. Additionally, calibration curves indicated that the nomogram could accurately predict one-, three-, and five-year OS (Figure 5C). A nomogram calibration curve was used to assess consistency between predicted and observed OS outcomes, with red, blue, and green lines indicating how the nomogram performed, whereas the gray line at 45° indicates flawless prediction (Figure 5D).

### 3.8 Gene set enrichment analysis and immune activity

**FIGURE 6:**
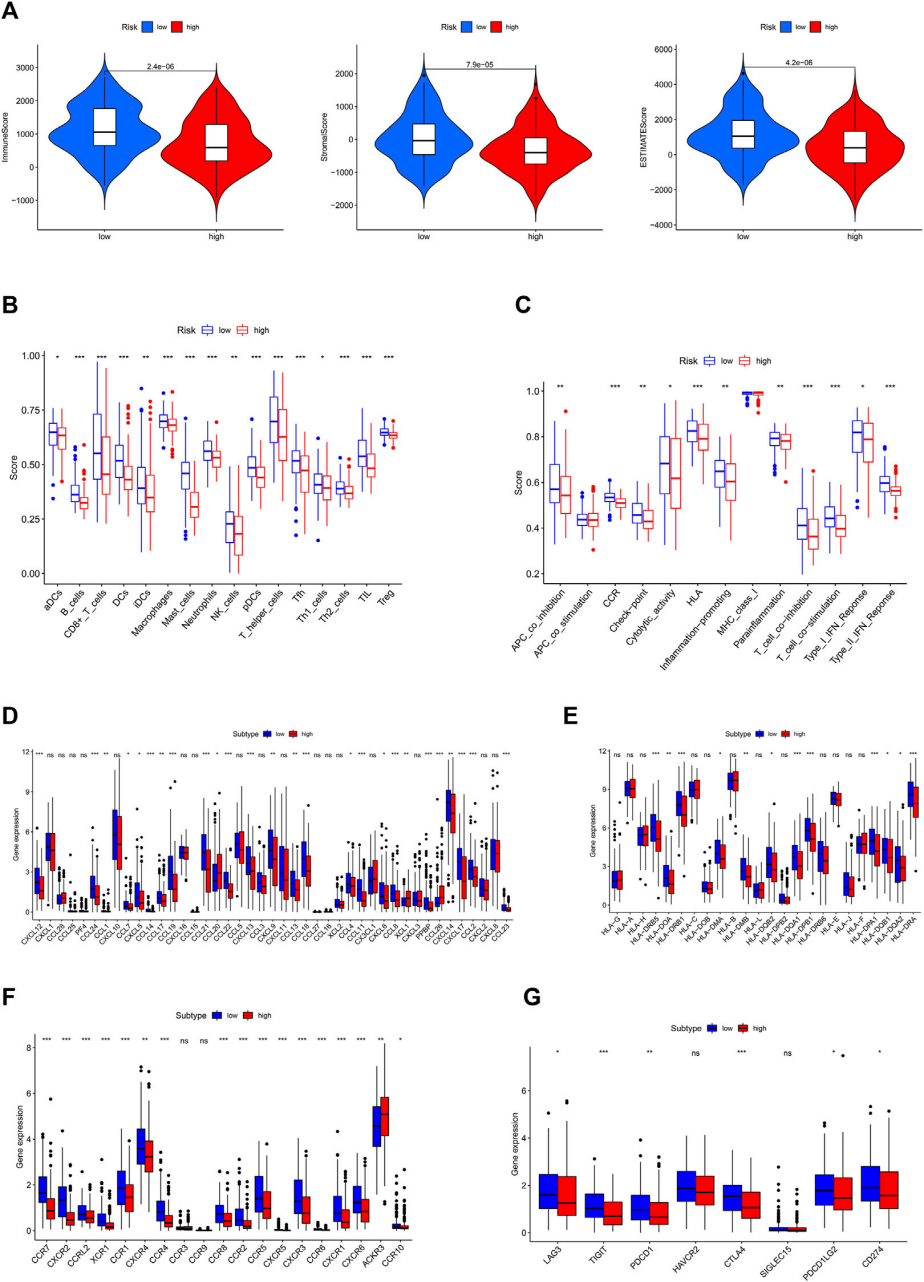
Two-group TME evaluation and checkpoints analysis. **(A)** Relationship between ESTIMATE score and high and low risk groups **(B)** The aggregation and expression of 16 immune cells were different in OSCC patients. **(C)** In high and low risk groups, 13 immunological functions expressed themselves differently. **(D)** Comparisons between the risk scores and the quantity of chemokines expression. **(E)** Human Leukocyte Antigen (HLA) expression in the high and low risk categories. **(F)** The abundance of chemokine receptors in different risk score groups. **(G)** Expression levels of clinically targetable ICP were measured in two risk groups. (*p* < 0.05 *; *p* < 0.01 **; *p* < 0.001 ***, not significant ns).

The ESTIMATE algorithm was applied to generate TME scores. This analysis showed that patients with high-risk scores had significantly lower estimate score, immune score, and stromal score (*p* < 0.001) than those patients with high-risk score (*p* < 0.001) (Figure 6A). Moreover, ssGSEA analysis of the differences in multiple immune cells and signal pathways revealed that the high-risk group had lower immune cell infiltration (*p* < 0.05; Figure 6B). Moreover, these pathways were suppressed in patients with high-risk scores, including APC co-inhabitation, CCR, immune checkpoint, and cytolytic activity (*p* < 0.05; Figure 6C). Chemokines mediate the leukocyte migration to various sites during normal homeostasis and inflammation. Therefore, we investigated the correlation between 19 chemokine receptors and 43 chemokines and risk categories (Figures 6D, F). This analysis revealed that most chemokines, including inflammatory chemokines like CCL2 and CXCL12, which promote the proliferation of B progenitor cells in the bone marrow milieu where they are produced, were markedly lower in patients with high-risk scores. Indicating that the differences between the immunological microenvironment of the high and low-risk groups were caused by the equivalent reduction in chemokine levels.

We also investigated the correlation between risk scores and HLA complex genes (Figure 6E), including HLA-DRB5 and HLA-DRB1, which are crucial for immune activity because of their antigen-presenting function. The potential of checkpoint inhibitors to treat cancer has attracted significant interest. Therefore, we deduced that the OSCC inflammatory condition may be associated with a unique expression of immune checkpoint (ICP). The expression levels of eight ICP genes,LAG3, TIGIT, PDCD1, HAVCR2, CTLA4, SIGLEC15, PDCD1LG2, and CD274 were calculated to determine the correlations between immune checkpoints and risk score. This analysis found LAG3, CTLA4, PDCD1, TIGIT, PDCD1LG2, and CD274 to be downregulated in the high-risk group (Figure 6G), suggesting that the low-risk group is sensitive to immunotherapy. To assess the response of patients to immune checkpoint inhibitors, we calculated the IPS scores of each sample and found that the IPS scores(ips_ctla4_pos_pd1_pos) of low-risk groups were higher, indicating that the patients in this group may be more sensitive to the combined PD-1/CTLA4 blockade (Supplementary Figures S6A–D).

### 3.9 Drug sensitivity analysis

**FIGURE 7:**
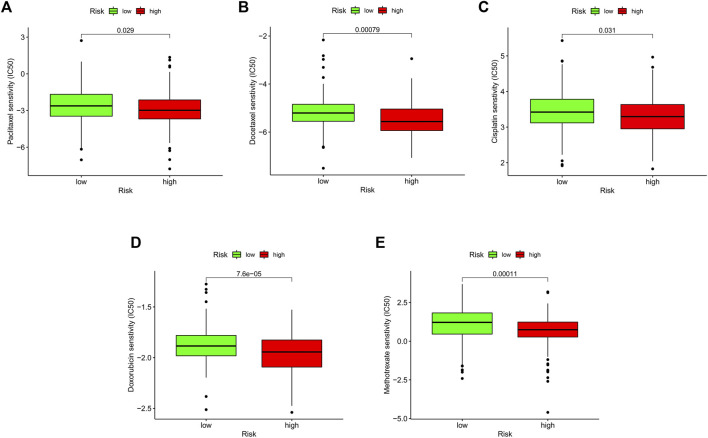
Relationship between risk score and therapeutic sensitivity. **(A–E)** Association between risk score and chemotherapeutic sensitivity.

Chemotherapy, targeted therapy, and immunotherapy may slow tumor growth in OSCC patients and enhance patient prognosis. We calculated the IC50 values of various chemotherapies in the test cohort using the “pRRophetic” package on R. This analysis found that paclitaxel, docetaxel, cisplatin, doxorubicin, methotrexate, and several targeted treatment drugs are more effective in patients with high-risk scores (Figures 7A–E). Paclitaxel primarily affects the M phase of mitosis, and disrupts tubulin synthesis, thereby inhibiting the replication of tumor cells. Docetaxel belongs to the same family as paclitaxel but has a higher affinity for microtubule sites and exhibits higher anticancer activity. Cisplatin is a platinum compound and acts on the chemical structure of DNA. Doxorubicin and methotrexate enter the nucleus, bind to DNA, and inhibit nucleic acid synthesis and mitosis. In summary, these findings suggest that risk scores can predict drug sensitivity.

## 4 Discussion

Because of its molecular heterogeneity, few treatments are effective against terminal oral cancer. To improve OSCC prognosis, novel biomarkers, and treatment targets are needed. The emergence of high-throughput array technologies presents a chance to investigate the mechanisms underlying OSCC occurrence and progression. Lysine acetylation, a key regulatory mechanism of gene expression, might be associated with OSCC pathophysiology but it is unclear if acetylation-related genes influence OSCC or whether they are associated with OSCC survival.

Here, we first assessed the expression levels of 33 LARGs in OSCC vs. normal tissues and found that most of were differentially expressed, with Sirtuin 5 (SIRT5) and Histone Deacetylase 3 (HDAC3) exhibiting the highest differential expression. Analysis of the correlation between the expression of SIRT5 and HDAC3 and overall survival revealed that both genes were linked to the prognosis of OSCC patients. HPA and RT-qPCR analysis of whether they are aberrantly expressed in OSCC showed that SIRT5 and HDAC3 were significantly upregulated in tumor tissues when compared with normal samples.

In OSCC, HDACs are thought to have excellent antitumor potential. It is proposed that RNA splicing and HDACs might be linked, with HDACs controlling acetylation and splicing through interaction with ribonucleoprotein complexes and the spliceosomes (Rahhal and Seto, 2019). Thus, we hypothesized that dysregulated acetylation might influence OSCC development by controlling RNA splicing. Lysine acetylation has been associated with the ribosome pathway, especially with the loss of acetylation on RPS6 and RPS3, which might have therapeutic target potential against OSCCs. (Dong et al., 2022). SIRT5 has been implicated in various malignancies. LDHA-K118su, a SIRT5 substrate markedly elevates invasion and migration by prostate cancer cells (Kwon et al., 2022). SIRT5 negatively regulates cancer cell proliferation in pancreatic ductal adenocarcinoma patients and is related to better prognosis. SIRT5 has also been associated with metabolic regulation and changes in the tumor microenvironment (Sun et al., 2022) in promoting hepatocarcinogenesis. SIRT5 deficiency can increase immune cell activity, indicating that it influences immune cell development (Wang K. et al., 2020).Our immune analyses indicate that acetylation influences the OSCC TME composition.

Next, two clusters were generated based on ‘HDAC3’ and ‘SIRT5’. To further evaluate the prognostic value of these acetylation-related regulatory factors, we used univariate and LASSO regression analyses to construct a risk model using eight genes and then validated its performance on an external dataset. We show that in OSCC patients, risk score is a reliable predictor of OS. Next, we developed a nomogram for clinical analysis of individualized prognosis and risk based on a risk score, age and stage. The calibration curve revealed a high fitness between the actual and predicted OS rates. Taken together, these findings indicate that the prognostic risk scoring model based on the eight-gene signature is an effective indicator of OSCC prognosis.

Next, we further investigated the eight genes used to construct the model. NK2 homeobox 3 (NKX2-3) has been reported as a prognostic factor in head and neck squamous cell carcinoma (HNSCC) (Huang L. et al., 2021; Liu et al., 2021). Suppressor APC domain containing neuroblastoma (SAPCD2) (Zhang et al., 2022), has been reported to regulate Yap/Taz, MAPK, and mTOR signaling in various cancers, including colorectal (Luo et al., 2020) and prostate cancer (Sun et al., 2021). Serine peptidase inhibitor Kazal type 7 (SPINK7) has also been proposed as a prognostic factor also a molecular biomarker in HNSCC (Pennacchiotti et al., 2021; Du et al., 2022). Ly6/neurotoxin 1 (LYNX1) has been suggested as a prognostic factor in ovarian serous cystadenocarcinoma (Liu et al., 2020) and glioblastoma (Ren et al., 2022). A quantitative sequencing study found that LYNX1 expression significantly increased the recurrence of methylation groups in oropharyngeal tumors. Aldo-keto reductase family 1 member C3 (AKR1C3) has been associated with poor prognosis in patients with oropharyngeal cancer, especially in HPV-positive patients (Peraldo-Neia et al., 2021). Synaptotagmin 17 (SYT17) was found to be differentially expressed in non-Hodgkin’s lymphoma (Fucà et al., 2021). MBL associated serine protease 1 (MASP1) has also been proposed as a prognostic factor in HNSCC and oral cancer (Belotti et al., 2021; Zhang and Wang, 2022). Cathepsin G (CTSG) overexpression is associated with poor diffuse large B-cell lymphoma survival (Carreras et al., 2021).

Numerous studies have found that the TME significantly influences cancer incidence, development, and metastasis (Belli et al., 2018; Laplane et al., 2019). Our analysis found that higher immune/stromal scores, were associated with lower risk scores, however, tumor purity had the opposite effect. In OSCC patients, higher risk scores predict a worse prognosis, which demonstrated that the higher the number of immune cells in OSCC, the more difficult it is to identify cancer cells (Gandara et al., 2018). The low infiltration level of antitumor immune cells indicates that immune function was impaired in the high-risk group (Li et al., 2017). Comparing the immune cell infiltration in high- and low-risk groups revealed that the number of invading immune cells in the high-risk group was less than in the low-risk group.

Intriguingly, we found that the proportion of Tregs was higher in the low-risk group than in the high-risk group. Tregs have been associated with subpar clinical outcomes and have been shown to downregulate anti-tumor immunity (Wolf et al., 2005; Toker et al., 2018). This might be explained by the need for Tregs in the TME to control excessive acetylation-induced inflammation Additionally, two key Treg subtypes identified in colon cancer have been shown to have competing roles in controlling the TME (Saito et al., 2016). The risk score was negatively associated with B cell infiltration. B cell infiltration in OSCC has not been extensively studied and available literature is inconsistent. B cell infiltration has been shown to enhance immunological function (Ammirante et al., 2010) while impairing T cell-dependent responses (Shalapour et al., 2015). Therefore, the different Treg subtypes in OSCC should be considered. Except for the APC co-stimulation pathway and MHC class I, the activities of other immunological pathways differed significantly between the two cohorts. These data suggest that a decrease in antitumor immunity may cause the low survival rates in high-risk OSCCs.

CCL2 is an important chemokine that is reported to promote the proliferation and metastasis of osteosarcoma cells by activating NF-κB signaling (Lazennec and Richmond, 2010; Chen et al., 2015). In the category of biological processes, the inflammatory reaction had the strongest correlation with risk scores. Inflammatory responses are reported to be crucial for cancer development, growth, malignant transformation, invasion, and metastasis (Tang et al., 2018). By controlling therapeutic response and immunological surveillance, inflammation also affects patient survival (Grivennikov et al., 2010).

Recent advances in bioinformatics have led to the development of powerful tools for identifying new cancer treatment targets, including for OSCC, based on tumor immunotherapy and microarray sequencing (Almangush et al., 2021; Huang G. G. et al., 2021). Although anti-PD-1/PD-L1 immunotherapy has been widely used to treat terminal OSCCs, only a limited number of cases benefit from this therapy (Dong et al., 2021). Hadler-Olsen et al. (Hadler-Olsen and Wirsing, 2019) discovered that CD163+ M2 and CD57^+^ showed a positive correlation with the outcome OSCC outcomes.

LAG3, TIGIT, PDCD1, CTLA4, PDCD1LG2, and CD274 checkpoints exhibit significant differences between patients with different risk scores. This may offer new immunotherapy strategies for OSCC and raises the possibility that patients in the high-risk category may benefit from ICP inhibitor treatment than patients with low-risk scores. In conclusion, our data indicate that immunosuppression might underlie poor prognosis in high-risk patients and that acetylation may be important for OSCC immunotherapy. However, this study has some limitations. First, the OSCC samples used are from public databases. Secondly, although our prognostic model has been confirmed in different datasets, the study is retrospective. To validate the clinical utility of the developed model, additional, well-designed studies are required. To determine the pathways involved, the identified genes should undergo experimental validation, either in cancer cells or mouse models. Additionally, we did not perform our own sequencing, and the follow-up data, as well as the sample size, were too small to carry out a similar survival study. We anticipate that the limitations highlighted above will define the scope and depth of our future research.

Few studies have examined the acetylation mechanisms underlying OSCC. Here, we identified two prognostic markers associated with acetylation in OSCC, SIRT5 and HDAC3, which are overexpressed in tumors, and found that their upregulation is associated with poor OS. We conducted a basic study on the prognostic value of these LARGs and built up some theoretical evidences to support future researches. The prognostic value of both genes warrants further validation using clinical data. Importantly, the prognosis model based on univariate Cox and LASSO regression analyses is closely associated with immune cell infiltration.

## Data Availability

The original contributions presented in the study are included in the article/Supplementary Material, further inquiries can be directed to the corresponding authors.
