# Supplementary material for: Integrative analysis of lysine acetylation-related genes and identification of a novel prognostic model for oral squamous cell carcinoma
Source: Front Mol Biosci. 2023 Aug 29;10:1185832. doi: 10.3389/fmolb.2023.1185832 (PMC10495994; doi:10.3389/fmolb.2023.1185832)
Supplement: Supplementary file 1 [file Table1.DOCX]

**Supplementary Materials：**

**Table S1** The clinical characteristics of OSCC patients from TCGA, and GSE41613.

| Factors |  | TCGA(n=323) | |  | GSE41613(n=97) | |
| --- | --- | --- | --- | --- | --- | --- |
| Age |  |  |  |  |  |  |
| ≤65 |  | 202 | 62.54% | ≤60 | 50 | 51.55% |
| >65 |  | 121 | 37.46% | >60 | 47 | 48.45% |
| Grade |  |  |  |  |  |  |
| G1-2 |  | 250 | 77.40% |  | N/A | |
| G3-4 |  | 65 | 20.12% |  |  |  |
| Gx |  | 6 | 1.86% |  |  |  |
| NA |  | 2 | 0.62% |  |  |  |
| Stage |  |  |  |  |  |  |
| Ⅰ-Ⅱ |  | 72 | 22.29% |  | 41 | 42.27% |
| Ⅲ-Ⅳ |  | 221 | 68.42% |  | 56 | 57.73% |
| NA |  | 13 | 4.02% |  |  |  |
| Gender |  |  |  |  |  |  |
| Male |  | 223 | 69.04% |  | 66 | 68.04% |
| Female |  | 100 | 30.96% |  | 31 | 31.96% |
| Vital status | |  |  |  |  |  |
| Dead |  | 147 | 45.51% |  | 51 | 52.58% |
| Alive |  | 176 | 54.49% |  | 46 | 47.42% |

| Gene |
| --- |
| CREBBP |
| EP300 |
| ESCO1 |
| ESCO2 |
| HAT1 |
| HDAC1 |
| HDAC10 |
| HDAC11 |
| HDAC2 |
| HDAC3 |
| HDAC4 |
| HDAC5 |
| HDAC6 |
| HDAC7 |
| HDAC8 |
| HDAC9 |
| HNF1A |
| KAT2A |
| KAT2B |
| KAT5 |
| KAT6A |
| KAT6B |
| KAT7 |
| KAT8 |
| LEF1 |
| SIRT1 |
| SIRT2 |
| SIRT3 |
| SIRT4 |
| SIRT5 |
| SIRT6 |
| SIRT7 |
| SLC16A10 |

**Table S2** Lysine acetylation-related genes used in this study.

**Table S3** Primers used in this study.

| Primers | Sequences | Product length |
| --- | --- | --- |
| HDAC3-F | 5’-ATTGGGCTGCTTTAACCTC-3’ | 118 bp |
| HDAC3-R | 5’-GGCAACATTTCGGACAGTA-3’ |  |
| SIRT5-F | 5’-CACCCTTGGCTTTTGTAGA-3’ | 124 bp |
| SIRT5-R | 5’-CTATGAGCATCCCCGTTC-3’ |  |
| GAPDH-F | 5’-GTCTCCTCTGACTTCAACAGCG-3’ | 131 bp |
| GAPDH-R | 5’-ACCACCCTGTTGCTGTAGCCAA-3’ |  |

**Table S4** Significant genes generated in Univariate Cox regression analysis

| Genes | Mean1 | Mean2 | logFC | pValue | fdr |
| --- | --- | --- | --- | --- | --- |
| SUSD4 | 6.87039 | 3.27932 | -1.06700 | 0.00469 | 0.00906 |
| STAG3 | 0.90054 | 0.44819 | -1.00668 | 0.00001 | 0.00003 |
| KRT1 | 281.31823 | 580.21620 | 1.04439 | 0.00008 | 0.00022 |
| CRNN | 29.47692 | 148.51937 | 2.33299 | 0.00005 | 0.00015 |
| ABHD11-AS1 | 0.96265 | 0.46943 | -1.03609 | 0.00006 | 0.00019 |
| CLDN8 | 1.67961 | 0.42469 | -1.98363 | 0.01307 | 0.02265 |
| NKX2-3 | 1.05126 | 0.27926 | -1.91247 | 0.00035 | 0.00088 |
| TMEM44-AS1 | 4.05648 | 1.96458 | -1.04601 | 0.00000 | 0.00000 |
| ELAPOR1 | 1.44869 | 0.63534 | -1.18916 | 0.00003 | 0.00010 |
| NKX1-2 | 0.80718 | 0.31493 | -1.35787 | 0.00000 | 0.00000 |
| NTS | 67.86085 | 4.13966 | -4.03500 | 0.00329 | 0.00661 |
| PI3 | 2054.62491 | 4312.53619 | 1.06966 | 0.00003 | 0.00009 |
| SHISA2 | 7.06479 | 3.52936 | -1.00124 | 0.00399 | 0.00782 |
| SOSTDC1 | 4.32883 | 2.00432 | -1.11086 | 0.02785 | 0.04413 |
| DEFB4B | 0.73704 | 3.41501 | 2.21207 | 0.00225 | 0.00470 |
| AL033397.1 | 1.95493 | 0.45674 | -2.09769 | 0.00028 | 0.00071 |
| AC012213.4 | 0.74624 | 0.31688 | -1.23570 | 0.00000 | 0.00000 |
| AC105118.1 | 0.83877 | 0.29951 | -1.48567 | 0.00218 | 0.00456 |
| H2BC17 | 1.14359 | 0.48209 | -1.24620 | 0.00000 | 0.00000 |
| NPPC | 2.16232 | 0.30524 | -2.82457 | 0.00002 | 0.00006 |
| SPDYC | 1.81868 | 0.73697 | -1.30322 | 0.00142 | 0.00309 |
| RASSF9 | 2.79272 | 1.19790 | -1.22116 | 0.00331 | 0.00664 |
| HAP1 | 1.99850 | 0.69626 | -1.52123 | 0.00000 | 0.00000 |
| CP | 3.21460 | 0.74875 | -2.10209 | 0.00703 | 0.01297 |
| C8G | 3.49121 | 1.68158 | -1.05391 | 0.00000 | 0.00000 |
| SOX2 | 33.42917 | 8.63037 | -1.95361 | 0.00003 | 0.00010 |
| UGT1A6 | 1.26124 | 0.33318 | -1.92047 | 0.00001 | 0.00004 |
| CENPV | 2.39667 | 1.08387 | -1.14485 | 0.00000 | 0.00000 |
| UGT1A7 | 2.73662 | 0.54446 | -2.32950 | 0.03038 | 0.04767 |
| CASC19 | 1.66756 | 0.67103 | -1.31329 | 0.00170 | 0.00364 |
| LINC01589 | 0.99699 | 0.49653 | -1.00570 | 0.00013 | 0.00036 |
| TOB1-AS1 | 1.85587 | 0.20174 | -3.20150 | 0.00000 | 0.00000 |
| KRT15 | 108.10729 | 50.47998 | -1.09868 | 0.00102 | 0.00228 |
| BIK | 13.02989 | 6.07883 | -1.09996 | 0.00000 | 0.00000 |
| TRIM16L | 7.09426 | 3.41782 | -1.05358 | 0.00015 | 0.00041 |
| LMO7-AS1 | 0.87618 | 0.39837 | -1.13712 | 0.00000 | 0.00000 |
| PLAC8 | 4.65638 | 1.14296 | -2.02644 | 0.00130 | 0.00285 |
| EDN2 | 10.07566 | 4.53603 | -1.15137 | 0.01185 | 0.02075 |
| IGLV4-69 | 14.50406 | 30.27416 | 1.06163 | 0.02148 | 0.03514 |
| NTRK2 | 7.42702 | 2.25142 | -1.72195 | 0.00100 | 0.00225 |
| AADAC | 0.94806 | 0.36011 | -1.39653 | 0.00603 | 0.01134 |
| TGM3 | 55.59872 | 138.25124 | 1.31417 | 0.00470 | 0.00909 |
| KRT6B | 1177.99604 | 2373.23429 | 1.01052 | 0.00000 | 0.00000 |
| MIR6835 | 0.77215 | 0.35163 | -1.13482 | 0.00000 | 0.00000 |
| WNK2 | 2.30346 | 0.60556 | -1.92746 | 0.00000 | 0.00000 |
| RBP7 | 4.24506 | 2.02640 | -1.06687 | 0.00068 | 0.00160 |
| WFDC12 | 14.07700 | 36.87792 | 1.38942 | 0.00001 | 0.00004 |
| KRT76 | 1.29152 | 3.55553 | 1.46100 | 0.00329 | 0.00662 |
| LINC01214 | 1.09870 | 2.26813 | 1.04570 | 0.00002 | 0.00007 |
| CEL | 8.38944 | 0.50322 | -4.05930 | 0.00000 | 0.00000 |
| AL109918.1 | 2.05651 | 0.46725 | -2.13792 | 0.00000 | 0.00000 |
| DSC1 | 5.31447 | 14.15500 | 1.41331 | 0.00008 | 0.00024 |
| CNFN | 261.18902 | 622.70591 | 1.25346 | 0.00000 | 0.00000 |
| PLLP | 1.29584 | 0.63563 | -1.02764 | 0.00000 | 0.00000 |
| NT5M | 1.46903 | 0.73312 | -1.00275 | 0.00000 | 0.00000 |
| SPINK5 | 32.33713 | 65.13343 | 1.01021 | 0.00059 | 0.00139 |
| LIPK | 1.30673 | 2.69286 | 1.04318 | 0.00005 | 0.00014 |
| IGKV1-13 | 0.30658 | 0.96751 | 1.65801 | 0.01891 | 0.03141 |
| SDR9C7 | 8.99347 | 18.48478 | 1.03939 | 0.00000 | 0.00000 |
| MMRN1 | 0.42857 | 0.97445 | 1.18505 | 0.00005 | 0.00015 |
| IGLV7-46 | 8.17316 | 18.61885 | 1.18780 | 0.03124 | 0.04882 |
| DLX6 | 0.94848 | 0.42192 | -1.16865 | 0.00002 | 0.00006 |
| SBK1 | 1.58660 | 0.57871 | -1.45502 | 0.00000 | 0.00000 |
| SLC34A2 | 2.18382 | 0.69656 | -1.64854 | 0.00890 | 0.01603 |
| MUC21 | 2.70239 | 9.46458 | 1.80830 | 0.00031 | 0.00079 |
| AGR2 | 12.06410 | 4.21567 | -1.51689 | 0.00693 | 0.01280 |
| PODXL2 | 9.65978 | 4.34032 | -1.15419 | 0.00002 | 0.00006 |
| SBSPON | 1.02534 | 0.35833 | -1.51673 | 0.00000 | 0.00001 |
| SLC5A1 | 2.17436 | 4.96006 | 1.18976 | 0.00001 | 0.00004 |
| LCE2D | 1.11106 | 2.26977 | 1.03061 | 0.00007 | 0.00021 |
| CDSN | 2.13363 | 4.59215 | 1.10586 | 0.00000 | 0.00002 |
| DLGAP1-AS2 | 0.99148 | 0.40945 | -1.27590 | 0.00000 | 0.00000 |
| PEG10 | 3.47328 | 1.69068 | -1.03870 | 0.00171 | 0.00367 |
| AP000251.1 | 0.80659 | 0.37498 | -1.10502 | 0.00000 | 0.00000 |
| AC005392.2 | 3.16257 | 6.51919 | 1.04359 | 0.00216 | 0.00452 |
| GSTA1 | 20.11402 | 1.04453 | -4.26728 | 0.00042 | 0.00102 |
| GPT | 0.92011 | 0.45177 | -1.02622 | 0.00000 | 0.00000 |
| VSIG10L | 6.80431 | 14.60418 | 1.10186 | 0.00000 | 0.00001 |
| ENDOU | 2.02109 | 5.95965 | 1.56010 | 0.00000 | 0.00000 |
| GSTA9P | 2.97290 | 0.21765 | -3.77176 | 0.01775 | 0.02971 |
| SYCP2 | 1.12397 | 0.47089 | -1.25513 | 0.00000 | 0.00001 |
| HAL | 0.65965 | 2.26202 | 1.77784 | 0.00015 | 0.00041 |
| PLEKHG4 | 3.65620 | 1.80265 | -1.02022 | 0.00000 | 0.00001 |
| SOX21 | 4.77601 | 2.22043 | -1.10496 | 0.01052 | 0.01865 |
| IGLV2-18 | 4.06023 | 8.83120 | 1.12105 | 0.01786 | 0.02988 |
| LEMD1 | 2.40233 | 1.16222 | -1.04755 | 0.00008 | 0.00022 |
| AC007906.2 | 2.42084 | 0.93768 | -1.36833 | 0.00071 | 0.00165 |
| CYP26A1 | 2.34294 | 0.38002 | -2.62417 | 0.00648 | 0.01206 |
| AC128709.2 | 0.68818 | 0.30849 | -1.15756 | 0.00344 | 0.00687 |
| AL139288.1 | 0.76313 | 0.37903 | -1.00961 | 0.00000 | 0.00000 |
| CASC8 | 1.28003 | 0.28043 | -2.19047 | 0.00000 | 0.00000 |
| RFC4 | 13.52217 | 6.74733 | -1.00294 | 0.00000 | 0.00000 |
| ABCC5 | 12.20726 | 5.50499 | -1.14893 | 0.00000 | 0.00000 |
| TGM1 | 57.20021 | 124.14140 | 1.11789 | 0.00000 | 0.00000 |
| MYB | 0.90584 | 0.41845 | -1.11419 | 0.00000 | 0.00000 |
| KRT19 | 355.88258 | 112.58899 | -1.66034 | 0.00014 | 0.00038 |
| AL391244.1 | 0.70049 | 0.29998 | -1.22350 | 0.00000 | 0.00000 |
| MELTF-AS1 | 2.28308 | 1.00118 | -1.18928 | 0.00000 | 0.00000 |
| RAB6B | 2.52061 | 0.60543 | -2.05773 | 0.00005 | 0.00017 |
| CHP2 | 2.57226 | 1.01581 | -1.34041 | 0.00189 | 0.00401 |
| PRR9 | 8.93208 | 21.66032 | 1.27799 | 0.00003 | 0.00011 |
| SLC16A14 | 1.34096 | 0.66919 | -1.00277 | 0.00011 | 0.00032 |
| SAPCD2 | 9.41675 | 4.67825 | -1.00926 | 0.00000 | 0.00000 |
| LINC00342 | 1.21843 | 0.59602 | -1.03159 | 0.00000 | 0.00000 |
| PRSS27 | 3.75855 | 7.52122 | 1.00079 | 0.00002 | 0.00007 |
| LINC00885 | 1.68680 | 0.52655 | -1.67965 | 0.00000 | 0.00000 |
| GPRC5D | 2.97784 | 1.23039 | -1.27515 | 0.00096 | 0.00218 |
| KRT78 | 8.83081 | 24.49129 | 1.47165 | 0.00000 | 0.00001 |
| COA6-AS1 | 2.18412 | 1.03060 | -1.08357 | 0.00000 | 0.00000 |
| OTX1 | 2.02161 | 0.92709 | -1.12473 | 0.00173 | 0.00370 |
| MUC4 | 2.62882 | 0.74375 | -1.82153 | 0.00665 | 0.01234 |
| GABRP | 8.12510 | 1.59947 | -2.34479 | 0.01109 | 0.01954 |
| LINC02870 | 0.91609 | 0.43093 | -1.08803 | 0.00000 | 0.00000 |
| CBWD4P | 0.71309 | 0.34497 | -1.04763 | 0.00000 | 0.00000 |
| DMRT2 | 1.36727 | 0.53912 | -1.34261 | 0.00654 | 0.01216 |
| COCH | 0.99709 | 0.21218 | -2.23247 | 0.00000 | 0.00002 |
| CXCR2 | 0.90651 | 1.90206 | 1.06916 | 0.00000 | 0.00000 |
| KRT2 | 4.21653 | 9.50693 | 1.17292 | 0.00005 | 0.00014 |
| YBX2 | 0.89343 | 0.31220 | -1.51687 | 0.00000 | 0.00000 |
| LINC00519 | 7.07181 | 2.67757 | -1.40116 | 0.00000 | 0.00000 |
| FGF19 | 0.99432 | 0.06346 | -3.96979 | 0.00076 | 0.00175 |
| FAM83E | 4.14777 | 2.02213 | -1.03646 | 0.00012 | 0.00033 |
| ZDHHC11B | 0.79839 | 0.32320 | -1.30466 | 0.00000 | 0.00000 |
| AL512329.2 | 0.78569 | 0.38741 | -1.02009 | 0.00005 | 0.00015 |
| A2ML1 | 26.72768 | 56.52131 | 1.08046 | 0.00000 | 0.00000 |
| MAFA-AS1 | 1.22031 | 0.37476 | -1.70321 | 0.00030 | 0.00076 |
| PANX2 | 5.13974 | 2.02686 | -1.34245 | 0.00000 | 0.00000 |
| KRT6C | 420.69018 | 1083.75593 | 1.36521 | 0.00000 | 0.00000 |
| PARD3-AS1 | 0.72480 | 0.30179 | -1.26404 | 0.00000 | 0.00000 |
| MAP7D2 | 1.28754 | 0.61647 | -1.06250 | 0.00548 | 0.01042 |
| AL021807.1 | 1.93402 | 0.95205 | -1.02249 | 0.00000 | 0.00002 |
| ALDH1A1 | 20.18570 | 3.95619 | -2.35115 | 0.00008 | 0.00023 |
| LINC01139 | 1.93909 | 0.85983 | -1.17326 | 0.00567 | 0.01073 |
| LINC02562 | 2.84415 | 1.41723 | -1.00492 | 0.00000 | 0.00001 |
| PLA2G4E | 9.46248 | 19.44196 | 1.03888 | 0.00000 | 0.00000 |
| LCE1F | 4.02115 | 8.58457 | 1.09414 | 0.01865 | 0.03103 |
| LCE2B | 1.92937 | 4.38903 | 1.18577 | 0.00014 | 0.00039 |
| RHCG | 96.85205 | 197.28288 | 1.02641 | 0.00212 | 0.00446 |
| CLTRN | 0.75483 | 0.37481 | -1.01000 | 0.00000 | 0.00000 |
| MRAP2 | 2.75796 | 0.63789 | -2.11222 | 0.00049 | 0.00118 |
| SPRR2A | 550.24960 | 1123.58838 | 1.02996 | 0.00002 | 0.00006 |
| SAMD12 | 1.58858 | 0.76716 | -1.05013 | 0.00140 | 0.00305 |
| MYO5C | 1.12938 | 0.48622 | -1.21586 | 0.00014 | 0.00038 |
| RIBC2 | 2.15735 | 0.97789 | -1.14151 | 0.00000 | 0.00000 |
| EPCAM | 28.18854 | 9.07983 | -1.63437 | 0.00000 | 0.00000 |
| KLK13 | 30.33071 | 65.39367 | 1.10837 | 0.00001 | 0.00003 |
| ELFN1-AS1 | 1.05815 | 0.44013 | -1.26553 | 0.00654 | 0.01216 |
| ACER1 | 0.85105 | 1.89318 | 1.15349 | 0.00033 | 0.00083 |
| AC091271.1 | 0.97419 | 0.48309 | -1.01191 | 0.00000 | 0.00000 |
| RPS2P32 | 0.91755 | 0.36267 | -1.33912 | 0.00012 | 0.00034 |
| SCUBE3 | 1.26376 | 0.31591 | -2.00013 | 0.00975 | 0.01741 |
| ALDH3A1 | 70.07886 | 26.18085 | -1.42047 | 0.00438 | 0.00851 |
| SCARNA13 | 0.72105 | 0.35923 | -1.00518 | 0.00000 | 0.00000 |
| C1QL1 | 2.43121 | 1.16669 | -1.05926 | 0.00001 | 0.00003 |
| SLC47A1 | 0.87883 | 0.42147 | -1.06016 | 0.00411 | 0.00803 |
| RPS3AP5 | 2.12428 | 0.94768 | -1.16451 | 0.00007 | 0.00021 |
| ULBP1 | 1.21017 | 0.34061 | -1.82901 | 0.00005 | 0.00017 |
| AL365181.3 | 2.75077 | 1.14381 | -1.26599 | 0.00009 | 0.00027 |
| CCDC190 | 2.16835 | 0.62117 | -1.80354 | 0.00051 | 0.00121 |
| SMTNL1 | 2.65533 | 1.00486 | -1.40190 | 0.01374 | 0.02366 |
| GLB1L2 | 1.13649 | 0.48205 | -1.23732 | 0.00005 | 0.00016 |
| C1QTNF12 | 5.80946 | 2.23821 | -1.37606 | 0.00096 | 0.00216 |
| SPINK7 | 12.47495 | 26.81365 | 1.10393 | 0.00003 | 0.00009 |
| NUPR2 | 1.53627 | 0.42867 | -1.84150 | 0.01029 | 0.01829 |
| LYPD5 | 6.94573 | 14.20216 | 1.03191 | 0.00000 | 0.00001 |
| PIR | 11.76095 | 5.59447 | -1.07193 | 0.00000 | 0.00000 |
| CYP2W1 | 1.45852 | 0.70066 | -1.05773 | 0.01177 | 0.02063 |
| SLC35G1 | 1.57921 | 0.53330 | -1.56619 | 0.00000 | 0.00000 |
| VSIG8 | 2.83838 | 8.46669 | 1.57673 | 0.00000 | 0.00001 |
| LINC01564 | 1.47532 | 0.40751 | -1.85611 | 0.00000 | 0.00000 |
| KRTCAP3 | 18.04847 | 9.00772 | -1.00264 | 0.00000 | 0.00000 |
| KCNMB2-AS1 | 2.31327 | 0.98881 | -1.22617 | 0.00000 | 0.00000 |
| FLG2 | 0.72598 | 1.85125 | 1.35051 | 0.00015 | 0.00040 |
| LINC00942 | 1.50539 | 0.39094 | -1.94512 | 0.00111 | 0.00247 |
| NPM2 | 1.09332 | 0.46765 | -1.22521 | 0.00000 | 0.00000 |
| TSPAN1 | 24.57330 | 12.14975 | -1.01617 | 0.00000 | 0.00001 |
| KPRP | 4.46420 | 13.74600 | 1.62254 | 0.00003 | 0.00010 |
| KRT16P1 | 1.37080 | 3.10536 | 1.17974 | 0.00002 | 0.00006 |
| RPL39L | 21.98551 | 10.75078 | -1.03211 | 0.00000 | 0.00000 |
| UGT8 | 1.84073 | 0.70221 | -1.39031 | 0.00000 | 0.00001 |
| CCDC146 | 1.49916 | 0.73653 | -1.02533 | 0.00000 | 0.00000 |
| SLC52A1 | 1.91340 | 0.89510 | -1.09602 | 0.00000 | 0.00000 |
| GPR160 | 1.94689 | 0.74643 | -1.38310 | 0.00010 | 0.00029 |
| MYL7 | 0.89132 | 0.19404 | -2.19958 | 0.00175 | 0.00375 |
| TH | 0.66681 | 1.73808 | 1.38215 | 0.00124 | 0.00274 |
| RBP4 | 1.19677 | 0.33781 | -1.82485 | 0.00390 | 0.00767 |
| AC112907.2 | 0.87897 | 0.35096 | -1.32450 | 0.00000 | 0.00000 |
| H1-4 | 1.24058 | 0.59465 | -1.06089 | 0.00000 | 0.00002 |
| TMEM52 | 1.41613 | 0.70524 | -1.00577 | 0.00002 | 0.00006 |
| COLEC11 | 0.76817 | 0.28867 | -1.41201 | 0.00186 | 0.00396 |
| LYNX1 | 15.82963 | 40.04717 | 1.33907 | 0.00000 | 0.00000 |
| GPX2 | 117.95059 | 42.22249 | -1.48210 | 0.00036 | 0.00090 |
| AC136475.3 | 1.76950 | 0.70635 | -1.32488 | 0.00000 | 0.00000 |
| PPP1R1B | 1.63464 | 0.46202 | -1.82293 | 0.00004 | 0.00012 |
| ADH1C | 2.85808 | 0.22551 | -3.66379 | 0.00001 | 0.00005 |
| MAL | 20.05257 | 52.30649 | 1.38320 | 0.00352 | 0.00701 |
| POMC | 2.40026 | 0.56137 | -2.09617 | 0.00012 | 0.00033 |
| KLK7 | 42.55139 | 86.38401 | 1.02156 | 0.00001 | 0.00005 |
| DMRTA2 | 1.24512 | 0.35251 | -1.82053 | 0.00006 | 0.00017 |
| SCGB1A1 | 5.21477 | 0.80434 | -2.69672 | 0.00720 | 0.01325 |
| PSPC1P1 | 2.06707 | 1.02365 | -1.01387 | 0.00222 | 0.00463 |
| BX293535.1 | 0.77717 | 0.29139 | -1.41528 | 0.00000 | 0.00000 |
| RNF222 | 1.59080 | 3.23910 | 1.02584 | 0.00000 | 0.00000 |
| S100A7A | 20.47977 | 46.50244 | 1.18311 | 0.00010 | 0.00028 |
| DACT2 | 1.96431 | 0.66716 | -1.55791 | 0.00133 | 0.00291 |
| IL36A | 5.61345 | 11.25998 | 1.00424 | 0.00099 | 0.00222 |
| CX3CL1 | 12.24926 | 5.14144 | -1.25245 | 0.00000 | 0.00000 |
| LINC01133 | 9.52140 | 4.46535 | -1.09240 | 0.00723 | 0.01330 |
| SLC29A4 | 1.40295 | 0.63769 | -1.13754 | 0.00566 | 0.01070 |
| CRCT1 | 52.54651 | 115.26762 | 1.13332 | 0.00000 | 0.00000 |
| VPS37D | 0.96447 | 0.45628 | -1.07983 | 0.00206 | 0.00434 |
| KLHL23 | 1.40610 | 0.55761 | -1.33438 | 0.00000 | 0.00000 |
| AC012615.1 | 1.32733 | 0.63287 | -1.06855 | 0.00000 | 0.00000 |
| RHOXF1-AS1 | 0.89413 | 0.13500 | -2.72752 | 0.00148 | 0.00321 |
| NLGN4X | 0.56130 | 1.26232 | 1.16924 | 0.00001 | 0.00002 |
| IL13RA2 | 1.01795 | 3.58659 | 1.81695 | 0.01435 | 0.02457 |
| CD70 | 4.08850 | 1.68170 | -1.28165 | 0.00036 | 0.00090 |
| LCE6A | 0.84570 | 2.02092 | 1.25679 | 0.00001 | 0.00003 |
| LRRC15 | 6.20110 | 12.55247 | 1.01738 | 0.00028 | 0.00072 |
| AC141557.1 | 1.22093 | 0.59956 | -1.02601 | 0.00003 | 0.00009 |
| NMRAL2P | 8.08041 | 1.79066 | -2.17394 | 0.00028 | 0.00071 |
| PKDCC | 2.34676 | 1.14403 | -1.03655 | 0.00097 | 0.00218 |
| AC020907.1 | 0.81336 | 0.25099 | -1.69626 | 0.00000 | 0.00000 |
| MSI1 | 1.26701 | 0.40441 | -1.64755 | 0.02302 | 0.03733 |
| ALOXE3P1 | 1.18827 | 4.10307 | 1.78784 | 0.00000 | 0.00001 |
| PRR15 | 1.51020 | 0.56966 | -1.40657 | 0.00000 | 0.00000 |
| SMKR1 | 0.90067 | 0.44025 | -1.03268 | 0.00027 | 0.00069 |
| LCE3D | 102.48218 | 209.19240 | 1.02946 | 0.00002 | 0.00008 |
| EIF2B5-DT | 0.66406 | 0.31999 | -1.05327 | 0.00000 | 0.00000 |
| AL512326.5 | 7.22953 | 3.26923 | -1.14495 | 0.00000 | 0.00000 |
| ABCA13 | 1.43083 | 0.63043 | -1.18244 | 0.00708 | 0.01305 |
| CLDN17 | 1.48662 | 4.08153 | 1.45707 | 0.00001 | 0.00002 |
| PLA2G4D | 1.16723 | 3.95332 | 1.75997 | 0.00000 | 0.00001 |
| PIPOX | 0.75932 | 0.36694 | -1.04917 | 0.00080 | 0.00185 |
| DSG1 | 43.50341 | 106.93921 | 1.29759 | 0.00000 | 0.00000 |
| PCDHGC5 | 0.62298 | 1.55789 | 1.32233 | 0.00000 | 0.00000 |
| PPBP | 0.83816 | 2.06743 | 1.30254 | 0.00012 | 0.00034 |
| AKR1C3 | 37.58042 | 12.22935 | -1.61963 | 0.02916 | 0.04597 |
| GPC6 | 1.12803 | 2.30214 | 1.02917 | 0.00051 | 0.00123 |
| RPRM | 0.90378 | 0.31264 | -1.53147 | 0.00000 | 0.00000 |
| KLK12 | 11.96724 | 29.08327 | 1.28110 | 0.00000 | 0.00001 |
| SCN9A | 0.98509 | 0.25672 | -1.94009 | 0.01402 | 0.02409 |
| ZDHHC2 | 2.32645 | 1.08640 | -1.09857 | 0.00001 | 0.00003 |
| SCEL | 11.34745 | 22.86800 | 1.01096 | 0.00001 | 0.00003 |
| PPARG | 0.80442 | 0.39183 | -1.03774 | 0.02589 | 0.04136 |
| LINC02561 | 0.82959 | 0.26826 | -1.62875 | 0.00051 | 0.00122 |
| METTL27 | 1.45936 | 0.66173 | -1.14103 | 0.00000 | 0.00000 |
| GSDMA | 6.24229 | 13.45731 | 1.10824 | 0.00000 | 0.00000 |
| SYT17 | 0.86924 | 0.38910 | -1.15962 | 0.00000 | 0.00000 |
| GFRA3 | 0.84587 | 0.20148 | -2.06977 | 0.00154 | 0.00333 |
| KLK14 | 20.76483 | 44.52859 | 1.10059 | 0.00014 | 0.00038 |
| NEURL1 | 1.17940 | 0.50766 | -1.21614 | 0.00010 | 0.00028 |
| STOX1 | 0.79892 | 0.24529 | -1.70359 | 0.00000 | 0.00000 |
| SPRR2C | 38.88414 | 79.05884 | 1.02375 | 0.00034 | 0.00084 |
| SPIRE2 | 0.96213 | 0.42666 | -1.17316 | 0.00000 | 0.00000 |
| SERPINI1 | 2.38739 | 1.04310 | -1.19456 | 0.01786 | 0.02988 |
| MTARC1 | 0.77951 | 0.33732 | -1.20847 | 0.00000 | 0.00000 |
| RPL23AP11 | 0.70073 | 0.34769 | -1.01106 | 0.02891 | 0.04561 |
| RCOR2 | 1.83771 | 0.84459 | -1.12159 | 0.00000 | 0.00002 |
| FSTL4 | 1.81658 | 0.87178 | -1.05918 | 0.00030 | 0.00076 |
| MSMB | 19.36175 | 3.89638 | -2.31300 | 0.00210 | 0.00440 |
| FNDC5 | 0.91517 | 0.43270 | -1.08068 | 0.01512 | 0.02575 |
| ALOX12B | 8.36427 | 18.42386 | 1.13926 | 0.00003 | 0.00008 |
| VTCN1 | 2.33423 | 0.67392 | -1.79231 | 0.00001 | 0.00005 |
| MORN3 | 0.71955 | 0.30931 | -1.21803 | 0.00001 | 0.00005 |
| AKR1C7P | 0.83274 | 0.32498 | -1.35752 | 0.00036 | 0.00090 |
| FOXA1 | 3.34432 | 1.12812 | -1.56779 | 0.00004 | 0.00012 |
| DEFB4A | 23.08245 | 55.98340 | 1.27820 | 0.00000 | 0.00001 |
| SMC1B | 0.77931 | 0.27268 | -1.51497 | 0.00001 | 0.00004 |
| VPREB3 | 0.85615 | 0.37061 | -1.20798 | 0.00010 | 0.00029 |
| PNCK | 1.70149 | 0.42108 | -2.01464 | 0.00000 | 0.00000 |
| S100A12 | 39.52167 | 86.54097 | 1.13074 | 0.00002 | 0.00006 |
| LIPN | 0.49771 | 1.10251 | 1.14743 | 0.00001 | 0.00004 |
| BPIFC | 1.26992 | 2.83567 | 1.15896 | 0.00000 | 0.00000 |
| RHPN1 | 2.24705 | 0.86926 | -1.37017 | 0.00000 | 0.00000 |
| H3C8 | 0.85860 | 0.35770 | -1.26321 | 0.00001 | 0.00003 |
| KRT8 | 72.39822 | 33.14405 | -1.12720 | 0.00000 | 0.00000 |
| SUSD2 | 1.62062 | 4.39091 | 1.43797 | 0.00926 | 0.01662 |
| KLHDC9 | 0.66647 | 0.32368 | -1.04198 | 0.00000 | 0.00001 |
| RPL21P13 | 1.44512 | 0.12699 | -3.50837 | 0.00000 | 0.00000 |
| TMEM116 | 1.34537 | 0.43610 | -1.62526 | 0.00000 | 0.00000 |
| AC108860.2 | 0.72985 | 0.32505 | -1.16692 | 0.00000 | 0.00000 |
| NR5A1 | 1.25698 | 0.49625 | -1.34083 | 0.01414 | 0.02428 |
| CLDN3 | 6.51671 | 1.69080 | -1.94644 | 0.00000 | 0.00001 |
| LCE3A | 16.07401 | 56.17157 | 1.80511 | 0.00000 | 0.00000 |
| SLC27A5 | 0.71319 | 0.33268 | -1.10013 | 0.00000 | 0.00000 |
| FOLR1 | 1.59847 | 0.68951 | -1.21304 | 0.01424 | 0.02443 |
| B4GALNT4 | 2.82709 | 1.01889 | -1.47232 | 0.00000 | 0.00001 |
| SULT2B1 | 19.73398 | 40.11312 | 1.02339 | 0.00000 | 0.00000 |
| UPK1B | 21.44764 | 4.71833 | -2.18447 | 0.02179 | 0.03557 |
| GCNT2 | 0.78069 | 0.32166 | -1.27922 | 0.00002 | 0.00008 |
| POU6F2-AS2 | 0.69312 | 0.32407 | -1.09679 | 0.02405 | 0.03878 |
| CABYR | 1.35796 | 0.60231 | -1.17286 | 0.00000 | 0.00000 |
| MUCL1 | 6.03493 | 14.39453 | 1.25411 | 0.00406 | 0.00795 |
| PRR4 | 0.83568 | 0.33385 | -1.32375 | 0.00000 | 0.00001 |
| AC005336.1 | 3.82872 | 1.11676 | -1.77755 | 0.02929 | 0.04614 |
| SLC27A2 | 1.23926 | 0.44246 | -1.48586 | 0.00003 | 0.00011 |
| PCOLCE2 | 1.31742 | 0.56060 | -1.23267 | 0.00035 | 0.00087 |
| GSTM2 | 1.51493 | 0.48458 | -1.64445 | 0.00043 | 0.00104 |
| SLURP1 | 44.42014 | 118.08681 | 1.41056 | 0.00001 | 0.00004 |
| GDF15 | 5.55131 | 1.93634 | -1.51950 | 0.00598 | 0.01124 |
| CAPS | 2.32801 | 1.06689 | -1.12569 | 0.00000 | 0.00000 |
| PAEP | 1.06012 | 2.15217 | 1.02157 | 0.00001 | 0.00005 |
| EPGN | 6.92682 | 14.19568 | 1.03519 | 0.00000 | 0.00000 |
| ARHGEF26 | 1.78188 | 0.53908 | -1.72483 | 0.00123 | 0.00271 |
| LINC01752 | 2.13112 | 1.02851 | -1.05105 | 0.00035 | 0.00088 |
| HEY1 | 6.34296 | 2.55830 | -1.30997 | 0.00001 | 0.00004 |
| LCE1A | 2.11167 | 4.92348 | 1.22130 | 0.00047 | 0.00114 |
| FGF14-AS2 | 0.67950 | 0.31697 | -1.10013 | 0.00009 | 0.00027 |
| BEX2 | 6.98087 | 2.32520 | -1.58605 | 0.00000 | 0.00000 |
| LCE2A | 1.66902 | 3.80356 | 1.18835 | 0.00031 | 0.00077 |
| MASP1 | 0.34486 | 0.72633 | 1.07461 | 0.00089 | 0.00202 |
| GLI1 | 1.32344 | 0.53765 | -1.29956 | 0.00691 | 0.01276 |
| CTSG | 0.92055 | 2.14397 | 1.21971 | 0.00003 | 0.00009 |
| LCE1D | 0.40954 | 0.93806 | 1.19569 | 0.00050 | 0.00119 |
| CHODL | 1.32690 | 0.57823 | -1.19835 | 0.00071 | 0.00166 |
| GPC3 | 10.60443 | 1.87802 | -2.49738 | 0.02155 | 0.03525 |
| LINC02298 | 2.16940 | 0.92343 | -1.23221 | 0.00000 | 0.00000 |
| AC083841.1 | 0.64448 | 1.74936 | 1.44062 | 0.00130 | 0.00286 |
| KRT84 | 0.70470 | 2.56507 | 1.86391 | 0.01740 | 0.02919 |
| FOXD3-AS1 | 1.14370 | 0.46705 | -1.29207 | 0.00000 | 0.00001 |
| FLG | 1.05699 | 3.33742 | 1.65877 | 0.00001 | 0.00002 |
| AK8 | 1.25272 | 0.58111 | -1.10819 | 0.00003 | 0.00011 |


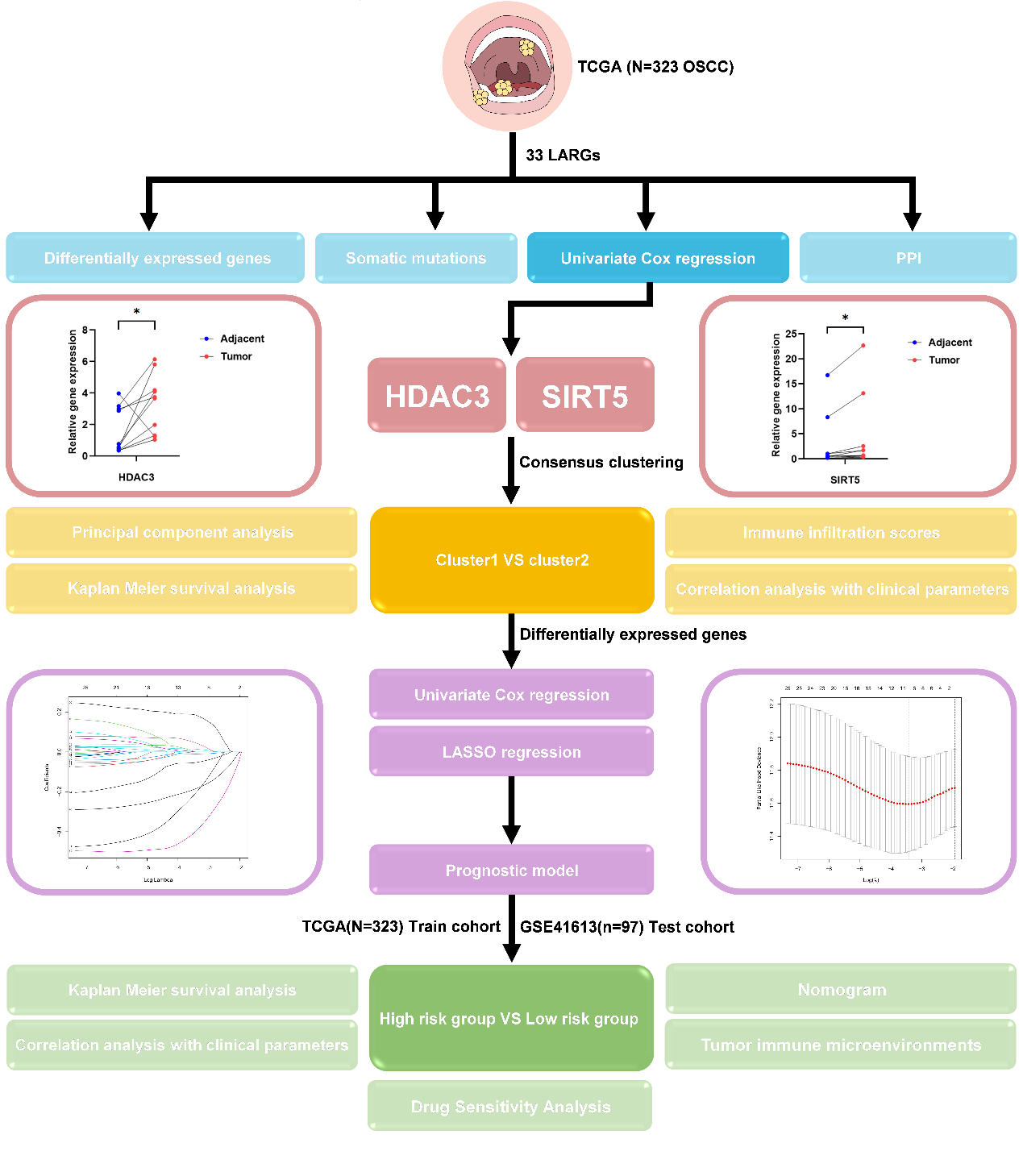


**Figure S1** Data and workflow for the prognostic analysis of OSCC with deep learning.


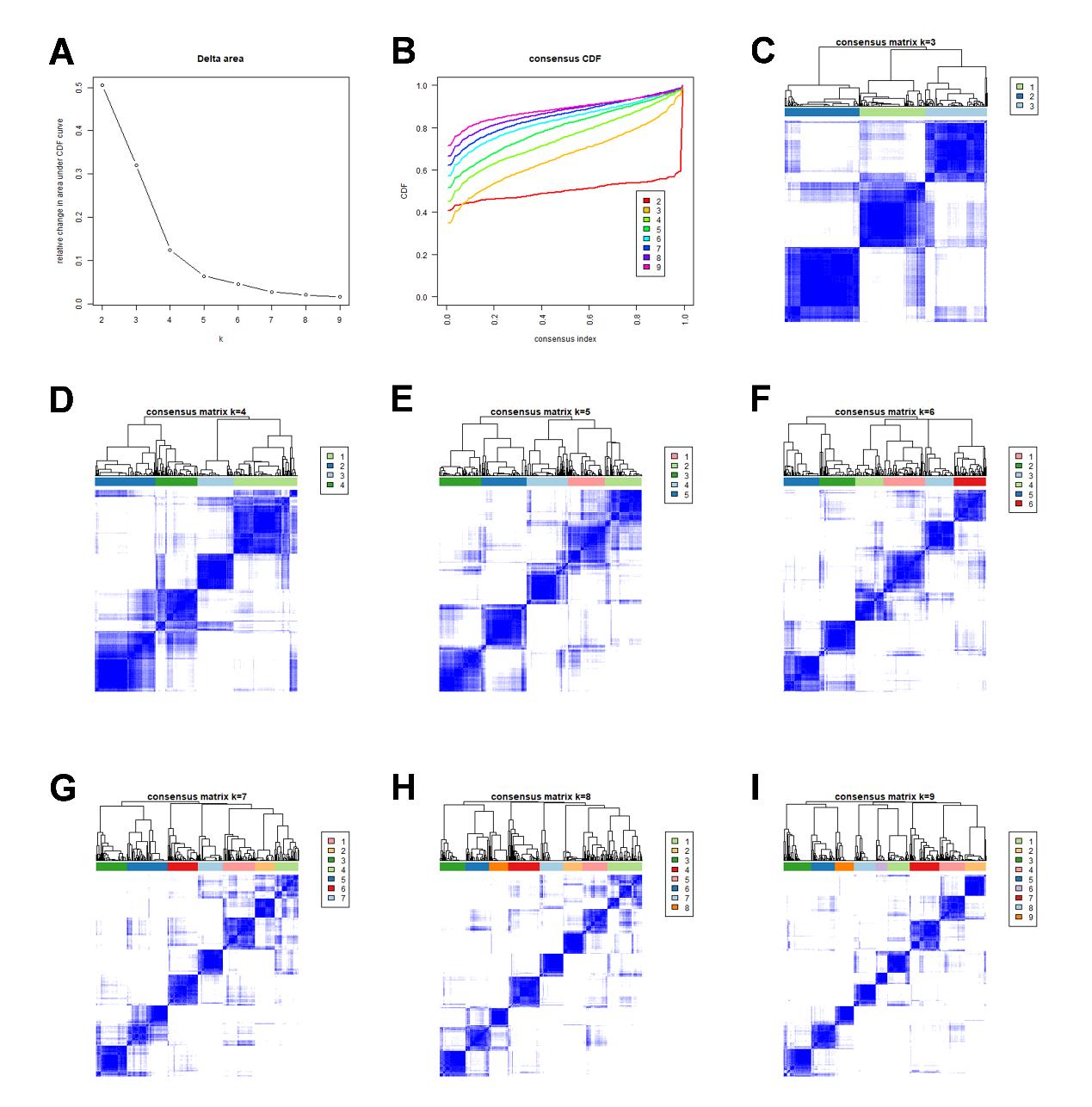


**Figure S2** Clusters of OSCC related to LARGs.

1. For k=2–9, relative changes in the region under the CDF are shown.
2. For k=2–9, an empirical CDF graph is shown.

(C-I) Using consensus clustering to divide OSCC samples into 3–9 groups.


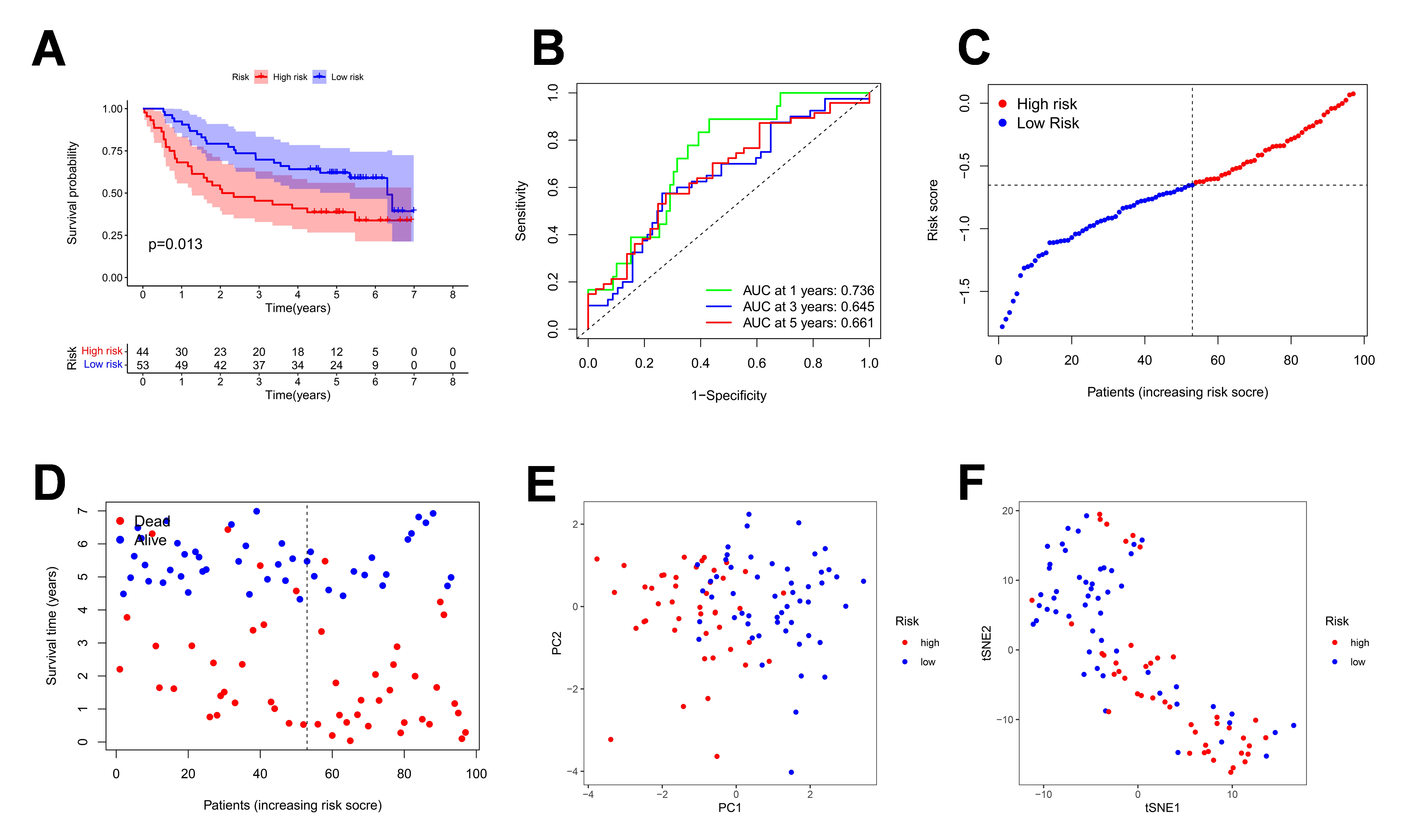


**Figure S3** Validation of the acetylation-related prognostic signature in the GEO cohort.

1. K-M curves showing the OS of OSCC patients in the high-risk group was significantly worse than that of the low-risk group.
2. ROC curves evaluate the predictive efficacy of the prognostic signature for OS in OSCC patients.

(C,D) Distribution of risk scores and survival status of patients in GEO cohort.

(E,F) Dimensionality reduction algorithms of PCA and t-SNE to show the samples of different ferroptosis-related risk groups were separately distributed.

**
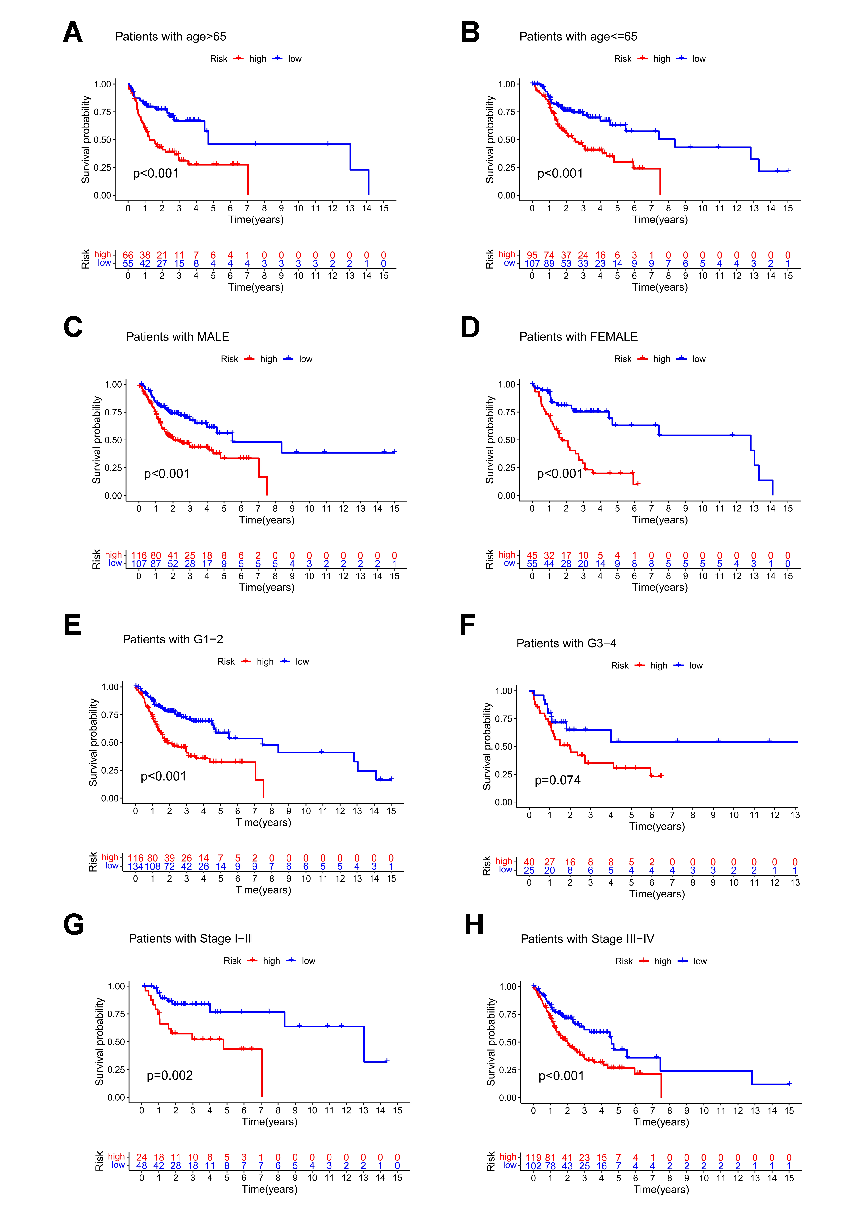
**

**Figure S4** Association between different clinical-groups and cancer prognosis.

(A–H) Kaplan–Meier analysis of OS in patients with age <= 65 (A) and age > 65 (B) in patients with male (C) and female (D) in patients with G1-2 (E) and G3-4 (F) in patients with Stage Ⅰ-Ⅱ (G) and Stage Ⅲ-Ⅳ (H).


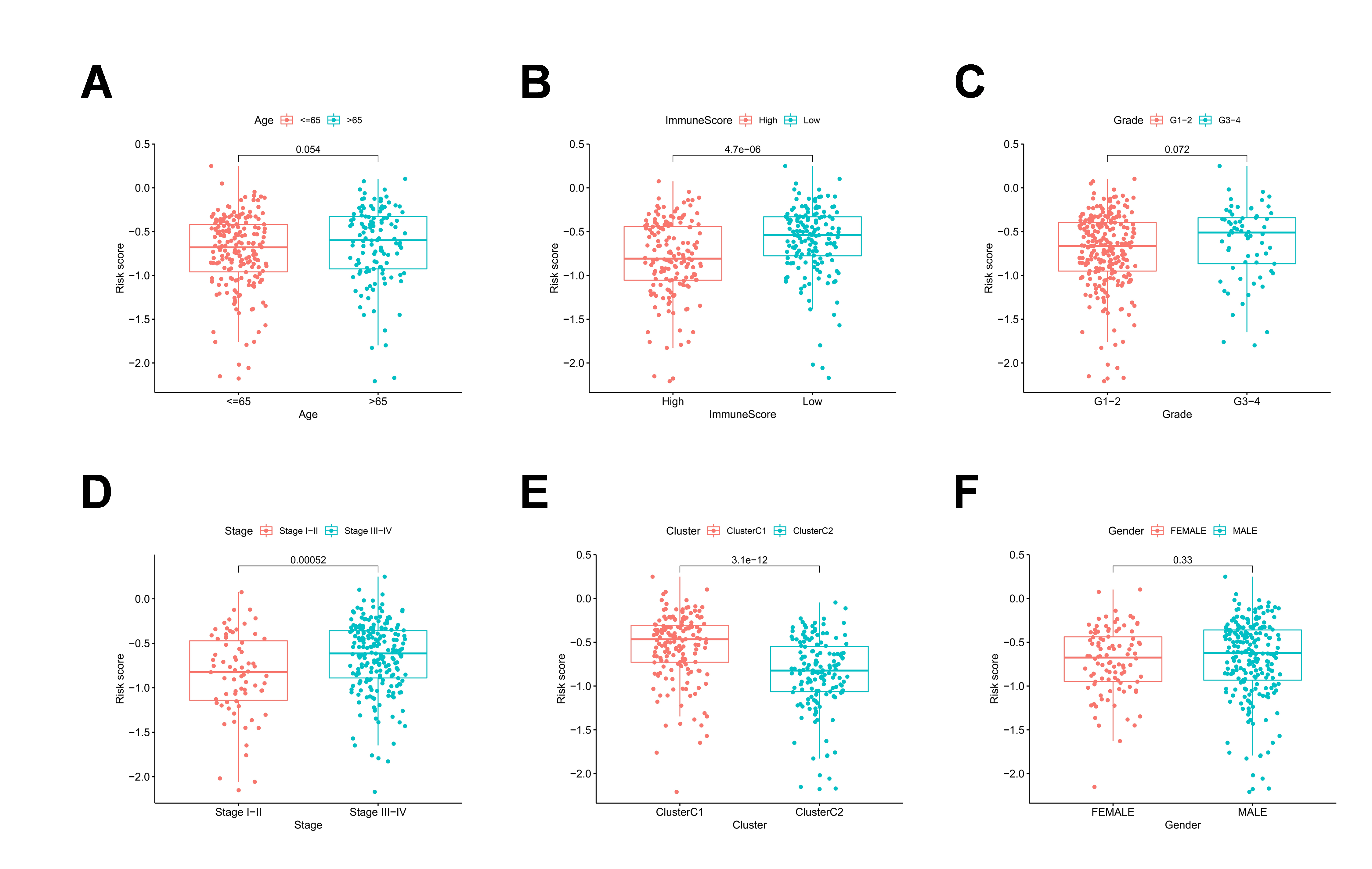


**Figure S5** Analysis of the statistical relationship between the risk score and the clinical indicators.

1. F) Relationship between risk score and age (A), immune score (B), grade (C), stage (D), clusters that we devided (E) and gender (F).

**
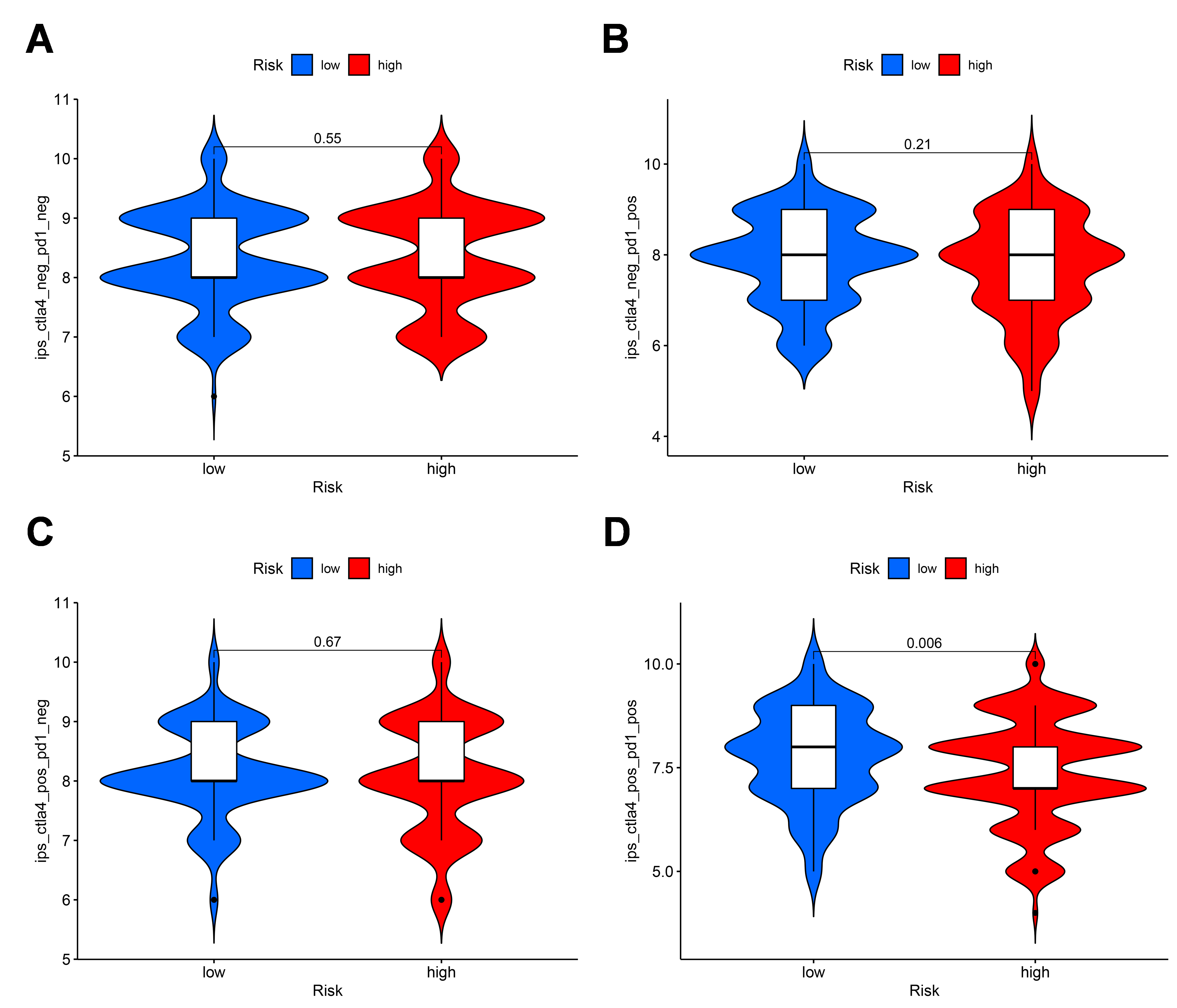
**

**Figure S6** Relationships between risk score and patients’ response to immunotherapy. (A–D) IPS in two risk groups.
